# Intrinsic RB activation induces tumoral and stromal anti-tumor responses that limit triple-negative breast cancer

**DOI:** 10.1038/s41523-025-00845-5

**Published:** 2025-12-01

**Authors:** Yin Wan, Jianxin Wang, Thomas N. O’Connor, Vishnu Kumarasamy, Ioannis Sanidas, Scott I. Abrams, Agnieszka K. Witkiewicz, Erik S. Knudsen

**Affiliations:** 1 Department of Molecular and Cellular Biology, Roswell Park Comprehensive Cancer Center, Buffalo, NY USA; 2 Krantz Family Center for Cancer Research, Massachusetts General Hospital, and Department of Medicine, Harvard Medical School, Boston, MA USA; 3 Department of Immunology, Roswell Park Comprehensive Cancer Center, Buffalo, NY USA

**Keywords:** Cancer, Cell biology

## Abstract

The RB tumor suppressor is a key regulator of cell cycle progression that is often inactivated in triple-negative breast cancer (TNBC). Recent studies indicate that drugs activating RB have multiple tumor-suppressing effects on the tumor and the tumor microenvironment (TME). Here, we utilize a constitutively active RB protein incapable of being phosphorylated and inactivated by CDKs (RBΔCDK) to assess the intrinsic sufficiency of RB activation on tumor suppression. Expression of RBΔCDK in TNBC cell lines uniformly inhibited proliferation. Transcriptomic analysis revealed suppression of cell cycle genes and the induction of genes associated with interferon response. Similarly, tumor growth and metastasis were suppressed in RBΔCDK-expressing human xenograft and mouse syngeneic tumor models. RB activation was sufficient to dramatically alter the TME, wherein tumor growth suppression was mediated by CD8^+^ T cells. Together, these data indicate that active RB suppresses TNBC progression in cancer cell-autonomous and non-autonomous mechanisms.

## Introduction

Triple-negative breast cancer (TNBC) is a highly aggressive subtype of breast cancer, associated with elevated recurrence rates and poor survival outcomes^1–3^. In contrast to other breast cancer subtypes, TNBC is characterized by complex genetic alterations and deregulated cellular proliferation, which impairs the efficacy of standard-of-care therapeutic strategies. Approximately 20-30% of TNBC cases exhibit mutation or loss of the retinoblastoma tumor suppressor (RB)^4–6^, a critical regulator of cell cycle progression. RB inactivation in TNBC often occurs through aberrant phosphorylation catalyzed by cyclin-dependent kinase (CDK)/Cyclin complexes^7–9^. Despite the success of CDK4/6 inhibitors in treating metastatic hormone receptor-positive breast cancer, these agents have shown limited efficacy in TNBC^10–13^, highlighting the need for novel therapeutic approaches.

In its canonical role, RB functions as a transcriptional co-repressor, preventing the expression of genes essential for cell cycle progression. This regulatory role is crucial for tumor suppression, as many of these genes are essential for cellular proliferation^14,15^. Consequently, strategies aimed at reactivating RB could offer broad therapeutic potential in cancers characterized by its loss or dysfunction^16^. However, resistance to CDK4/6 inhibitors is often driven by mechanisms that inactivate RB or dysregulate its phosphorylation through parallel pathways, such as CDK2/Cyclin E^17–21^. This has led to the exploration of combinatorial strategies that restore RB activation or prevent its phosphorylation in an effort to enhance the sensitivity of tumor cells to CDK4/6 inhibitors and suppress their proliferative capacity^22–26^.

Beyond its role in cell cycle control, recent studies have highlighted the ability of CDK4/6 inhibitors to reprogram the tumor microenvironment (TME)^10,27^. In various tumor models, CDK4/6 inhibition has been shown to induce a gene expression signature resembling that of interferon-stimulated genes, which is associated with enhanced immunological responses. As CDK4/6 inhibition has systemic effects and pleiotropic roles in cellular processes, it remains unclear whether these immune-modulatory effects are directly linked to tumor intrinsic RB activation. Given that the interferon response is often associated with robust immune infiltrates, there is growing evidence suggesting that CDK4/6 inhibitors can synergize with immune checkpoint inhibitors (ICIs) to enhance anti-tumor immunity and improve therapeutic outcomes^27–29^. These studies (that include preclinical TNBC models) suggest that the combination of cell cycle inhibition and immune modulation may hold therapeutic promise. Moreover, in clinical settings, neoadjuvant CDK4/6 inhibition has been shown to induce an interferon-like response, alongside suppression of cell cycle progression in hormone receptor-positive (HR+)/human epidermal growth factor receptor 2-negative (HER2-) breast cancer^27,30^.

In this study, we aimed to investigate the sufficiency of RB activation in controlling tumor growth and metastasis in models that are largely resistant to CDK4/6 inhibitors. We also sought to explore the tumor intrinsic role of RB activation in modulating the immune landscape of TNBC to differentiate from the systemic effects imparted by treatment with CDK4/6 inhibitors.

## Results

### Expression of active RB inhibits cell growth in palbociclib-resistant TNBC cell lines

**Fig. 1:**
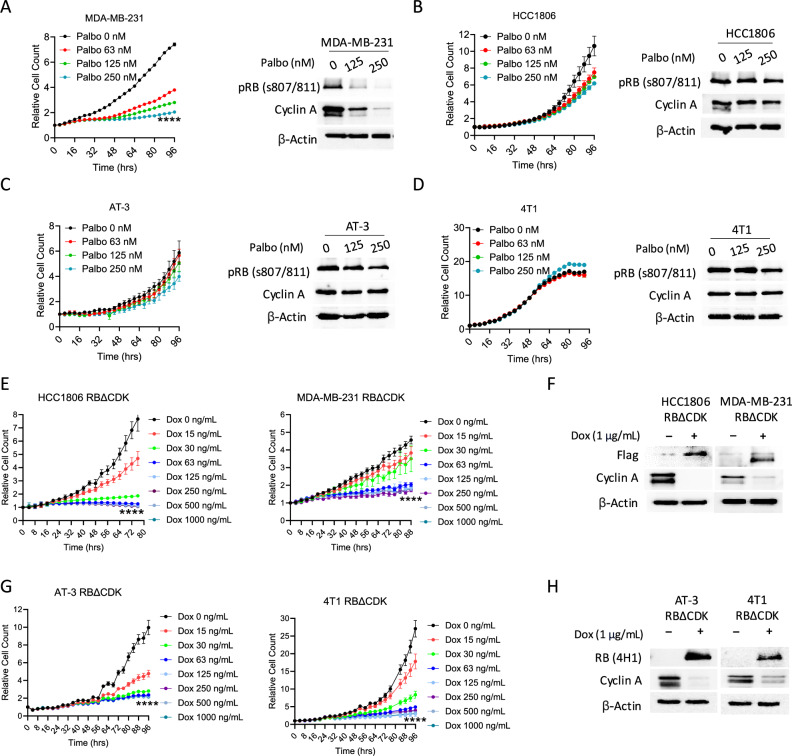
RB activation inhibits cell growth in palbociclib-resistant TNBC cell lines. **A**–**D** Live cell imaging using IncuCyte S3 and subsequent immunoblotting on the indicated cell lines treated with different concentrations of palbociclib. Cell proliferation was determined based on GFP positivity counts. Immunoblotting was performed on the indicated cell lines treated with different concentrations of palbociclib for 48 h. **E** Live cell imaging of the RBΔCDK HCC1806 and MDA-MB231 cell lines treated with different concentrations of Doxycycline (Dox). **F** Immunoblotting of the indicated RBΔCDK human cell lines in the presence and absence of Dox. **G** Live cell imaging of the indicated RBΔCDK murine cell lines treated with different concentrations of Dox. **H** Immunoblotting of the indicated RBΔCDK murine cell lines in the presence and absence of Dox. Data reported as mean +/− SEM, *n* = 4 for each live-cell cohort, ****p < 0.0001 as determined by two-tailed *t*-test.

To define the impact of RB activation, we examined the cellular responses of multiple TNBC cell lines to the CDK4/6 inhibitor, palbociclib, which pharmacologically activates RB as its primary mechanism of action^31^. The human TNBC cell line MDA-MB-231 showed significant proliferative arrest upon palbociclib treatment, as determined by live-cell imaging (Fig. 1A). Biochemical analysis revealed that the palbociclib-mediated growth arrest was associated with blockade of RB phosphorylation and repression of cyclin A, which is a critical cell cycle regulatory protein downstream from RB (Fig. 1A). In contrast, growth of the human TNBC cell line HCC1806 and the murine TNBC cell lines, AT-3 and 4T1, were largely unaffected by palbociclib at concentrations up to 1 μM (Fig. 1B–D, S1A). Consistent with the lack of growth inhibition in these resistant models, RB phosphorylation and Cyclin A expression remained unchanged after palbociclib treatment (Fig. 1B–D).

To determine whether cell growth was dependent on RB phosphorylation, a doxycycline (Dox)-inducible system was used to express Flag-tagged human wild-type RB or a mutant RB allele that is incapable of being phosphorylated at any of the 14 in vivo CDK phosphorylation sites (RBΔCDK) (Fig. S1B–D)^32^^,33^. The transduced cells were subjected to clonal selection to produce homogeneous cell populations that express the RBΔCDK isoform, which was validated based on Flag expression determined by immunofluorescence (IF) (Fig. S1B). The impact of RBΔCDK expression on cell proliferation was then assessed using live-cell imaging. In HCC1806 and MDA-MB-231 cells, expression of RBΔCDK led to a significant reduction in cellular growth (Fig. 1E). Biochemical analysis also confirmed that RBΔCDK expression suppressed Cyclin A expression (Fig. 1F). Similarly, in AT-3 and 4T1 cells, proliferation was suppressed with RBΔCDK expression (Fig. 1G, S1C, D), and Cyclin A levels were reduced (Fig. 1G, H). Expression of exogenous, Dox-inducible wildtype RB (RB-WT) in 4T1 cells in the presence and absence of Dox was then assessed. Expression of RB-WT was confirmed by immunofluorescent (IF) staining with Dox treatment (Fig. S1E). Live-cell imaging revealed that 4T1 cells expressing RB-WT and treated with either Dox or vehicle displayed pronounced cell proliferation in vitro (Fig. S1F). To validate RB function in human TNBC models, we ectopically expressed RB-WT in HCC1806 and MDA-MB-231 cells. Successful expression of RB-WT was confirmed by both IF staining (Fig. S1G, I) and biochemical assays (Fig. S1K). Similar to the observations in 4T1 cells, RB-WT–expressing HCC1806 and MDA-MB-231 cells exhibited robust in vitro proliferation (Fig. S1H, J) and expression of the cell cycle progression marker Cyclin A (Fig. S1K) upon treatment with either doxycycline or vehicle control, suggesting that ectopically expressed RB-WT is quickly phosphorylated and inactivated. Further, vehicle and Dox treated non-transduced MDA-MB-231 and HCC1806 cells exhibited robust in vitro proliferation and served as an important treatment control (Fig. S1L). Overall, these findings demonstrate that activation (and not simply expression) of RB is sufficient to impede cell cycle progression and proliferation in palbociclib-resistant TNBC models in a cancer cell-autonomous manner.

### Expression of active RB is sufficient to impact multiple transcriptional programs in TNBC cell lines

**Fig. 2:**
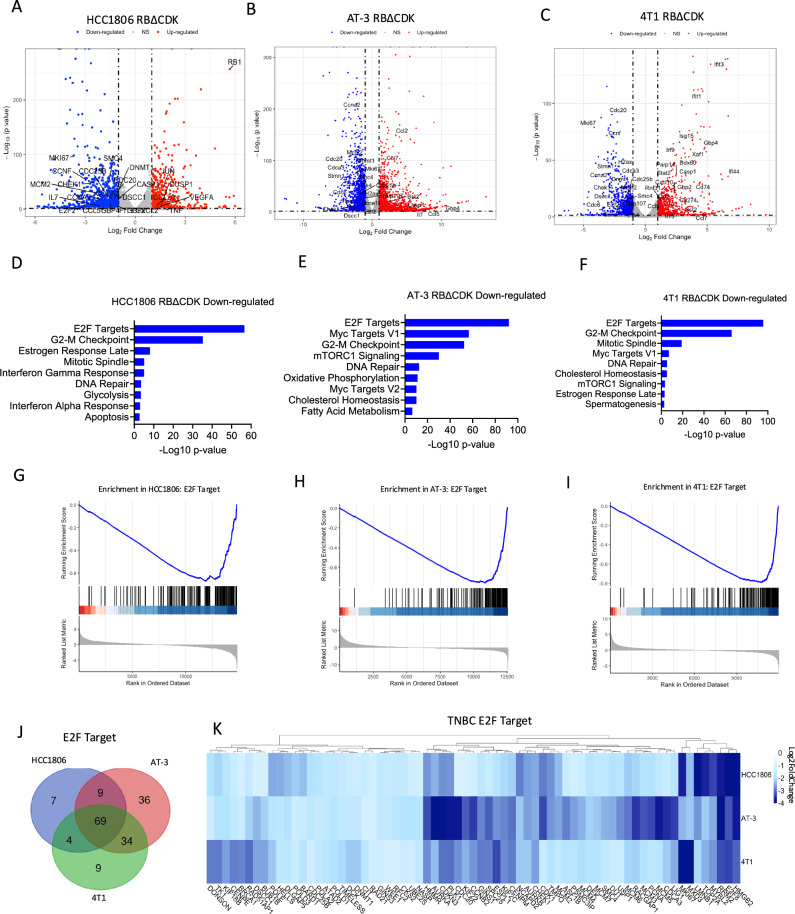
RB activation impacts transcriptional programs in TNBC cell lines. **A**–**C** Volcano plots indicating the differentially expressed genes based on transcriptome analysis in the indicated RBΔCDK cell lines treated with Doxycycline (Dox) (1 μg/mL) for 48 hours compared to non-treated cells. Blue represents genes that were significantly downregulated, red represents genes that were significantly upregulated. **D**–**F** ENRICHR analysis of the most significant downregulated pathways from the indicated cell lines following RBΔCDK activation. **G**–**I** Gene set enrichment analysis (GSEA) of the E2F Target pathway from the indicated cell lines following RBΔCDK activation. **J** Venn diagram depicting the overlap of significantly downregulated E2F target genes from the indicated cell lines following RBΔCDK activation. **K** E2F target pathway gene expression heatmap as Log_2_ fold change from the indicated cell lines following RBΔCDK activation.

To investigate the effects of RB activation at the transcript level, RNA sequencing (RNA-seq) was performed on four different TNBC models with Dox-inducible RBΔCDK constructs (HCC1806, AT-3, 4T1 and MDA-MB-231) in the absence and presence of Dox. Transcripts with an adjusted *p*-value < 0.05 and Log_2_ fold change > 1 or < −1 were deemed to be significantly altered. Activation of RBΔCDK resulted in substantial gene expression changes across all cell lines, suggesting sufficiency of RB in regulating the transcriptional programs (Fig. 2A–C)^33^. In RBΔCDK-activated HCC1806, AT-3, and 4T1 models, 1969, 2404 and 1070 genes, respectively, were found to be significantly downregulated. ENRICHR analysis demonstrated that among the most significantly downregulated pathways following RBΔCDK activation across all tested cell lines were the E2F target and G2-M checkpoint pathways (Fig. 2D–F). Gene Set Enrichment Analysis (GSEA) further validated the significant downregulation of E2F target genes with RBΔCDK expression across all four cell lines (Fig. 2G–I, S2A–E). Direct assessment of downregulated E2F target genes revealed that 69 genes were consistently and significantly downregulated across RBΔCDK-activated TNBC cell lines (Fig. 2J, K). Collectively, these results underscore that RBΔCDK activation induces robust cell cycle arrest by downregulating critical transcripts within the RB/E2F pathway in TNBC models.

**Fig. 3:**
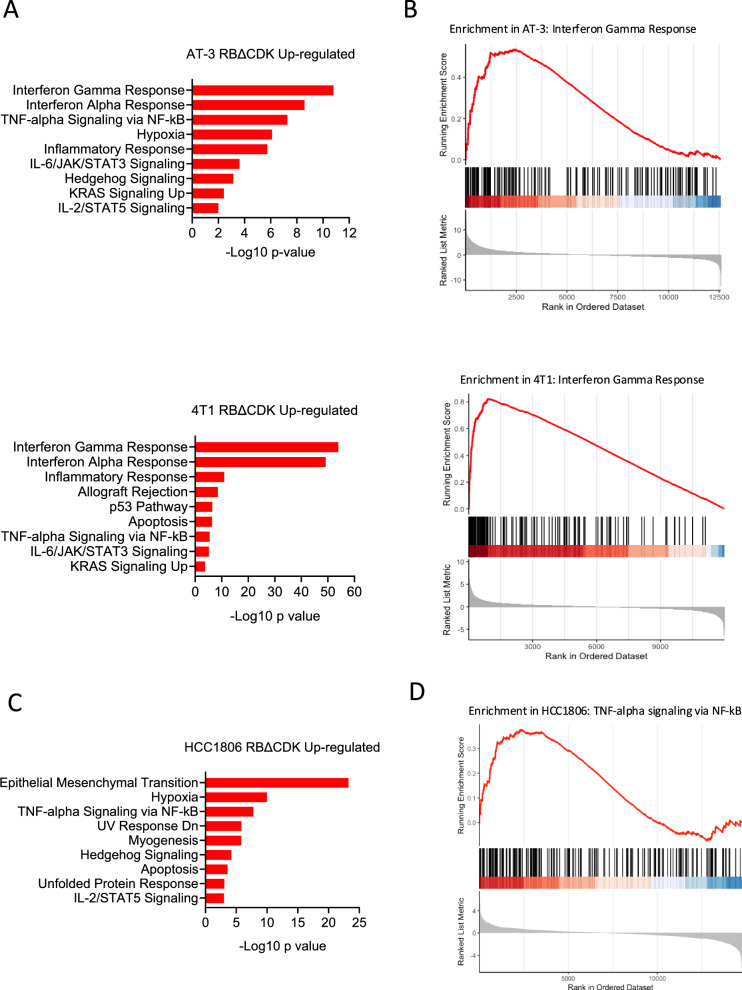
RB activation upregulates immune response genes in TNBC cell lines. **A** ENRICHR pathway analysis of significantly upregulated genes from the indicated cell lines following RBΔCDK activation with doxycycline compared to non-treated controls. **B** Gene set enrichment analysis (GSEA) of the Interferon Gamma Response pathway from the indicated cell lines following RBΔCDK activation. **C** ENRICHR pathway analysis of significantly upregulated genes in the HCC1806 cell line following RBΔCDK activation. **D** GSEA of the TNF-alpha Signaling via NFκB pathway from the HCC1806 cell line following RBΔCDK activation.

Previous studies have indicated that RB1 activation may play a role in transcriptionally repressing epigenetic modifiers, such as DNMT1 and EZH2, which are involved in suppressing endogenous retroviruses and the subsequent IFN responses triggered by dsRNA^27,34,35^. In both AT-3 and 4T1 models, the expression of RBΔCDK led to the induction of 2785 and 1143 genes, respectively (Fig. 2B, C). Pathway analysis of these upregulated transcripts revealed that interferon-gamma (IFNγ) and interferon-alpha (IFNα) signaling pathways were most notably elevated in both cell lines following RBΔCDK activation (Fig. 3A). These results were further supported by GSEA, which showed a significant enrichment of IFNγ response genes in RBΔCDK-activated AT-3 and 4T1 cells (Fig. 3B). In contrast, RBΔCDK-activated HCC1806 cells, both ENRICHR and GSEA analyses identified a marked enhancement of TNFα/NFκB signaling among the 1283 significantly upregulated genes (Fig. 3C, D). However, RBΔCDK-activated MDA-MB-231 cells displayed no substantial gene upregulation (Fig. S2D). Together, these findings demonstrate that RB activation is sufficient to influence gene expression relevant to immune system activity in select TNBC contexts.

To further elucidate the transcriptional impact of RBΔCDK activation in vivo, we integrated RNA-seq data from both AT3 and 4T1 murine TNBC models expressing RBΔCDK constructs. This analysis revealed 38 commonly upregulated and 100 commonly downregulated genes across both models (Fig. S3A). Gene ontology enrichment indicated that the upregulated genes were primarily involved in immune-related pathways, including antigen presentation and interferon signaling, while the downregulated genes were associated with cell cycle progression, DNA replication, and mitotic checkpoints. To assess the clinical relevance of these findings, we applied the RBΔCDK-regulated gene signature to the METABRIC breast cancer dataset. Unsupervised clustering based on the expression of these genes identified three distinct patient groups (Fig. S3B). Kaplan-Meier survival analysis demonstrated a strong prognostic association: patients in Cluster 3, characterized by high expression of immune response genes and low expression of cell cycle-related genes, trended to have better overall survival as compared to patients in Cluster 2 that displayed suppressed immune signaling and elevated cell cycle gene expression (Fig. S3C). These findings suggest that RBΔCDK-induced transcriptional reprogramming may stratify tumors by immunogenicity and proliferative potential, with implications for prognosis and therapeutic response.

### TNBC 3D culture and in vivo tumor growth are blunted upon RB activation

**Fig. 4:**
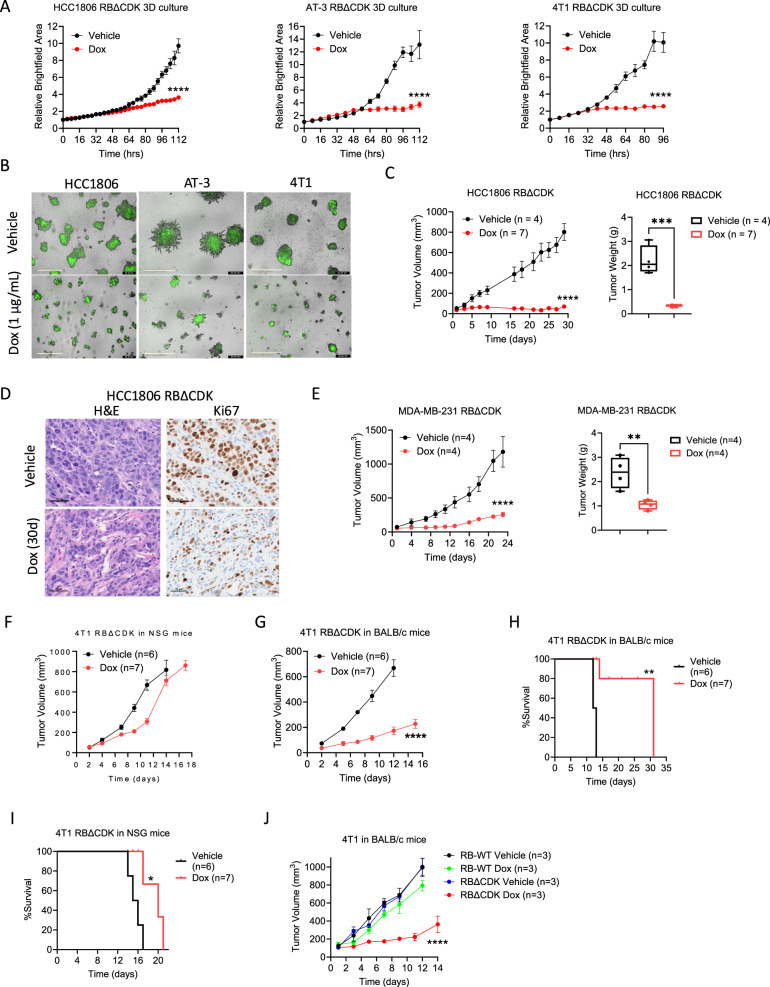
TNBC 3D culture and in vivo tumor growth is blunted upon RB activation. **A**, **B** Live cell proliferation curves from 3D cultures of the indicated RBΔCDK TNBC cell lines in the presence and absence of doxycycline (Dox) (1 μg/mL) as measure by GFP-positive counts with representative images. Scale bar = 400 μm. Error bars represent mean +/− SEM from two separate experiments that were done in triplicate. ****p < 0.0001 as determined by two-tailed *t*-test. **C** Tumor volume (plotted as mean +/− SEM) and box and whisker plots of tumor mass of RBΔCDK HCC1806-derived xenografts after 30 days following vehicle or Dox water (2 mg/mL) treatment. ***p < 0.001, ****p < 0.0001 as determined by two-tailed *t*-test. **D** Hematoxylin and eosin (H&E) and immunohistochemical staining for Ki67 expression of excised RBΔCDK HCC1806-derived xenografts between vehicle and Dox water-treated (2 mg/mL) mice from **C**. Scale bar = 50 μm. **E** Tumor volume (plotted as mean +/− SEM) and box and whisker plots of tumor mass of RBΔCDK MDA-MB-231-derived xenografts following vehicle or Dox water (2 mg/mL) treatment. **p < 0.01, ****p < 0.0001 as determined by two-tailed *t*-test. **F** Tumor volume of RBΔCDK 4T1-derived xenografts in NSG mice following vehicle or Dox water (2 mg/mL) treatment. **G** Tumor volume of RBΔCDK 4T1-derived tumors in BALB/c mice following vehicle or Dox water (2 mg/mL) treatment. ****p < 0.0001 as determined by two-tailed *t*-test. Data displayed as mean +/− SEM. **H** Survival curves of BALB/c mice harboring RBΔCDK 4T1-derived tumors treated with vehicle or Dox water (2 mg/mL). **p < 0.01 as determined by log-rank test. **I** Survival curves of NSG mice harboring RBΔCDK 4T1-derived xenografts treated with vehicle or Dox water (2 mg/mL). *p < 0.05 as determined by log-rank test. **J** Tumor volume of RBΔCDK 4T1-derived xenografts in NSG mice following vehicle or Dox water (2 mg/mL) treatment ****p < 0.0001 as determined by two-way ANOVA. Data displayed as mean +/− SEM.

Given that RBΔCDK activation inhibits TNBC cell growth in 2D culture, we next explored its effects in 3D culture derived from the HCC1806, AT-3, and 4T1 cell lines. In these 3D cultures, RBΔCDK activation resulted in a significant reduction in growth across all three models (Fig. 4A, B). To further assess the impact of RBΔCDK activation in vivo, we conducted xenograft experiments with HCC1806 cells that were injected into the mammary fat pad of immunodeficient NSG mice. The mice were divided into two groups: one receiving vehicle and the other Dox to induce RBΔCDK expression. Activation of RBΔCDK resulted in pronounced disease control, with significantly reduced tumor volume and mass compared to the vehicle-treated group (Fig. 4C, S4A). Immunohistochemical (IHC) analysis of tissue excised from HCC1806 xenograft tumors confirmed that RBΔCDK activation reduced staining of the proliferation marker Ki67 (Fig. 4D). Similarly, in MDA-MB-231 xenografts in NSG mice, RBΔCDK activation resulted in a significant reduction in tumor volume and mass, as compared to mice treated with vehicle (Fig. 4E, S4B).

Although RBΔCDK activation inhibited 4T1 growth in 3D culture, in vivo efficacy of RBΔCDK activation was modest in limiting 4T1-derived tumor growth in NSG mice. Dox treatment appeared to reduce tumor growth over the first 10 days; however, tumors quickly resumed growth despite Dox exposure (Fig. 4F). Given the pronounced upregulation of interferon signaling observed in RBΔCDK-activated 4T1 cells compared to RBΔCDK-activated HCC1806 and MDA-MB-231 cells, 4T1 cells were implanted, and growth was evaluated in syngeneic immune-competent female BALB/c mice. In this context, Dox-treated mice harboring 4T1 RBΔCDK-activated tumors displayed significantly reduced tumor growth over 15 days (Fig. 4G). IHC staining of excised 4T1-derived tumors from BALB/c mice validated the expression of RBΔCDK (Fig. S4C). In accordance with the significant disease control, RBΔCDK activation prolonged the survival of BALB/c mice with a median overall survival of 31 days, compared to 12 days for vehicle-treated mice (Fig. 4H). As expected, immune-deficient NSG mice displayed modest benefit of RBΔCDK activation with respect to median overall survival (Fig. 4I). The impact of RBΔCDK activation on AT-3 tumor growth was also assessed in both immunocompetent (C57BL/6NCrl) and immunodeficient (NSG) mouse models under continuous Dox treatment. However, RBΔCDK activation failed to elicit a detectable anti-tumor response in either setting (Fig. S4D, S4F). IHC staining revealed modest expression of RB in both NSG and C57BL/6NCrl mouse models and relatively unchanged levels of the proliferation markers Ki67 and cyclin A between vehicle and Dox-treated mice, indicating a lack of cell cycle suppression in AT-3 tumors (Fig. S4E, S4G).

The impact of expressing exogenous Dox-inducible wildtype RB (RB-WT) and RBΔCDK on 4T1 syngeneic tumor growth was then assessed. IHC staining of excised 4T1-derived tumors confirmed the expression of RB-WT (Fig. S4H). RB-WT expression in 4T1 syngeneic model did not significantly affect tumor growth in vivo (Fig. 4J, S4I). Similarly, overexpressing RB-WT in HCC1806 xenografts had a modest impact on tumor growth, as compared to RBΔCDK expression (Fig. S4J). Collectively, these findings suggest that RB activation, rather than mere overexpression, is responsible for the suppression of tumor growth in vivo, with the immune response potentially playing a key role in sustaining disease control.

### RB activation limits metastatic potential of TNBC in vivo

**Fig. 5:**
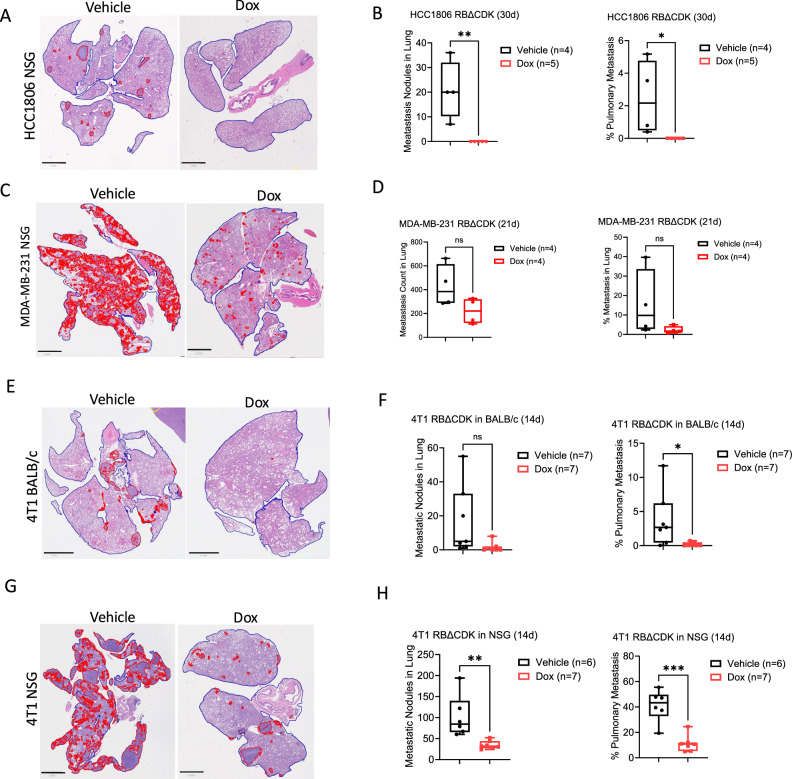
RB activation limits metastatic potential of TNBC tumors. **A** Representative hematoxylin and eosin (H&E) images of lung tissue excised from NSG mice implanted with RBΔCDK HCC1806 xenografts treated with either vehicle or doxycycline (Dox) water (2 mg/mL) for 30 days. Red demarcations denote metastasis nodules. Scale bar = 2 mm. **B** Box and whisker plots of metastases and tumor outgrowth in the lungs of NSG mice implanted with RBΔCDK HCC1806 xenografts treated with either vehicle or Dox water (2 mg/mL) for 30 days. **C** Representative H&E images of lung tissue excised from NSG mice implanted with RBΔCDK MDA-MB-231 xenografts treated with either vehicle or Dox water (2 mg/mL) for 21 days. **D** Box and whisker plots of metastases and tumor outgrowth in the lungs of NSG mice implanted with RBΔCDK MDA-MB-231 xenografts treated with either vehicle or Dox water (2 mg/mL) for 21 days. **E** Representative H&E images of lung tissue excised from BALB/c mice implanted with RBΔCDK 4T1 tumors treated with either vehicle or Dox water (2 mg/mL) for 14 days. **F** Box and whisker plots of metastases and tumor outgrowth in the lungs of BALB/c mice implanted with RBΔCDK 4T1 tumors treated with either vehicle or Dox water (2 mg/mL) for 14 days. **G** Representative H&E images of lung tissue excised from NSG mice implanted with RBΔCDK 4T1 xenografts treated with either vehicle or Dox water (2 mg/mL) for 14 days. Scale bar = 2 mm. **H** Box and whisker plots of metastases and tumor outgrowth in the lungs of NSG mice implanted with RBΔCDK 4T1 xenografts treated with either vehicle or Dox water (2 mg/mL) for 14 days. *p < 0.05, **p < 0.01, ***p < 0.001 as determined by two-tailed *t*-test.

TNBC tumors frequently exhibit distant metastatic spread with disease progression^36^. Therefore, we examined the impact of RB activation on spontaneous pulmonary metastasis in NSG mice harboring HCC1806 xenograft-derived tumors. Hematoxylin and eosin (H&E) staining revealed a significant reduction in pulmonary tumor outgrowth in RBΔCDK-activated tumor-bearing mice compared to vehicle-treated controls, as evidenced by a lower number of metastatic lesions and reduced overall lung tumor burden (Fig. 5A, B). Similar trends were observed in MDA-MB-231 xenografts, although RBΔCDK activation did not result in significant decreases in lung tumor outgrowth (Fig. 5C, D).

Consistent with the in vivo tumors derived from human cell lines, RBΔCDK activation significantly reduced the percentage of metastatic lesions in the lungs compared to vehicle-treated animals in 4T1 tumors isolated from syngeneic BALB/c mice (Fig. 5E, F). As a complementary approach to validate the anti-metastatic impact of RB activation, an experimental metastasis model involving tail vein injections of 4T1 cells was implemented in BALB/c mice, followed by Dox or vehicle treatment. Dox-treated mice exhibited significantly fewer lesions in the lungs, reinforcing the role of RB activation in suppressing pulmonary metastasis in this TNBC setting (Fig. S4A, B). Interestingly, although RBΔCDK activation had a modest impact on 4T1 primary tumor growth in immune-deficient NSG mice, H&E staining revealed a marked reduction in spontaneous pulmonary metastases in the RBΔCDK-activated group compared to controls in NSG mice (Fig. 5G, H). Trans-well migration assays using TNBC cell lines demonstrated that RBΔCDK activation did not significantly alter the invasive or migratory capacity of the cells in vitro (Fig. S4C). In conclusion, these findings suggest that the suppression of metastasis may be driven by a mechanism distinct from its control of primary tumor growth and may not be entirely dependent on the host immune system.

### RB activation in TNBC tumor models alters the tumor microenvironment

**Fig. 6:**
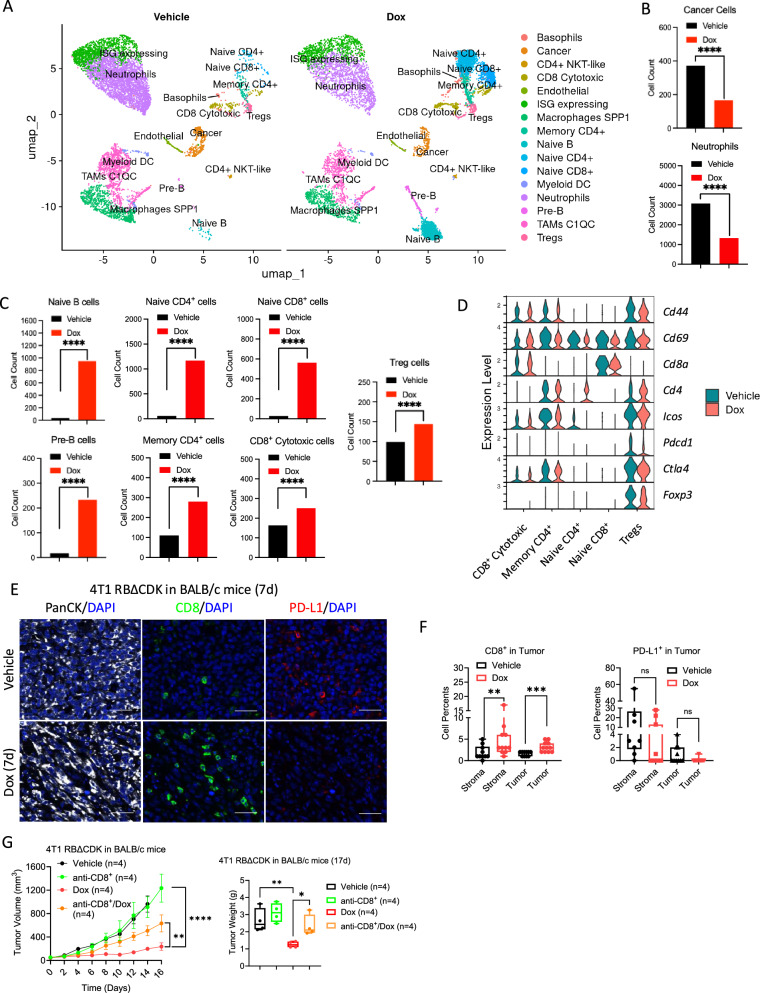
RB activation in TNBC tumor models alters the tumor microenvironment. **A** Single-cell RNA sequencing cluster analysis of RBΔCDK 4T1 tumors derived from BALB/c mice treated with either vehicle (left) or doxycycline (Dox) water (2 mg/mL) (right) for 6 days. **B**, **C** Quantification of cell types from various clusters identified in **A**. **D** Transcript expression violin plots from T cell clusters identified in **C**. **E** Representative multispectral immunofluorescent (mIF) images of RBΔCDK 4T1 tumors from BALB/c mice treated with either vehicle or Dox water (2 mg/mL) for 7 days stained for pan-cytokeratin (PanCK, white), CD8 (green), PD-L1 (red), and DAPI (blue). Scale bar = 50 μm. **F** Box and whisker plots quantifying CD8 and PD-L1 mIF image staining from tumors in E in both the tumor and stromal compartments. **p < 0.01, ***p < 0.001 as determined by two-tailed *t*-test, *n* ≥ 3 mice per condition, with analysis from 3–5 regions of interest (ROIs) per mouse. **G** Tumor volume (plotted as mean +/- SEM) and box and whisker plots of tumor mass of RBΔCDK 4T1 tumors in BALB/c mice treated with vehicle, Dox water (2 mg/mL), anti-CD8 antibody, and the combination of Dox water and anti-CD8 antibody. *p < 0.05, **p < 0.01, ****p < 0.0001 as determined by two-way ANOVA.

To further investigate the role of the immune system in RBΔCDK-mediated tumor suppression, the tumor microenvironment (TME) was examined in immunocompetent BALB/c mice bearing RBΔCDK-activated 4T1-derived tumors. Single-cell RNA sequencing of tumors excised from Dox or vehicle-treated mice identified an array of immune and tumor cell populations (Fig. 6A, S6A, B). Significant alterations in cell populations were observed following RBΔCDK activation; tumor cell and neutrophil populations declined, while B and T cell populations increased, along with upregulation of *Cd4* and *Cd8* in the T cell cluster (Fig. 6A–C). Further analysis of the CD8^+^ cytotoxic cell population compared with the naïve CD8^+^ T cell population revealed increased expression of *Cd44*, *Cd69*, and *Icos*, which are markers of T cell activation, and minimal changes were observed in the immune checkpoint markers *Pdcd1* and *Ctla4* (Fig. 6D). We also investigated the abundance of regulatory T cells (Tregs) between the vehicle and Dox-treated mice. While Tregs appeared to slightly increase with Dox treatment, violin plots revealed that the expression of Treg markers *Foxp3* and *Ctla4* was slightly reduced in Dox-treated mice (Fig. 6C, D). Multispectral immunofluorescence staining of excised 4T1 tumors from both RBΔCDK-activated and control-treated mice was then conducted to validate observations from the single-cell RNA sequencing analysis. Consistently, RBΔCDK-activated tumors and the surrounding stroma exhibited a substantial increase in CD8^+^ T cell infiltration based upon CD8 staining (Fig. 6E, F). Additionally, while not found to be significant, RBΔCDK activation trended to reduce PD-L1 staining, suggesting that RB activation in the tumor compartment could potentially enhance the anti-tumor immune response by reducing immune exhaustion (Fig. 6E, F). While the expression of CD3 and FOXP3 trended to increase with Dox treatment in the stromal compartment, no significant changes were observed (Fig. S6C, D). Similarly, no significant changes were observed with treatment when comparing CD11c or CD163 expression, consistent with the lack of observed changes in the M1 and M2 macrophage populations by single-cell RNA sequencing analysis (Fig. S6C, D).

To assess the functional significance of CD8^+^ T cell infiltration in RBΔCDK-mediated tumor suppression, BALB/c mice bearing 4T1 RBΔCDK tumors were treated with vehicle, Dox, anti-CD8^+^ antibody, or a combination of Dox and anti-CD8^+^ antibody. Treatment with anti-CD8^+^ antibody significantly blunted the inhibition of primary tumor growth with RB activation (Fig. 6G), further supporting the crucial role of CD8^+^ T cells in mediating the anti-tumor effects upon RB activation. Potential synergies between RBΔCDK activation and ICIs in controlling tumor progression were also explored. BALB/c mice were treated with vehicle, Dox, Dox plus anti-PD-L1, or Dox plus anti-CTLA-4. However, no significant differences in tumor progression were observed between the RBΔCDK-activated groups with or without ICIs (Fig. S6E). These results suggest that, while RB activation modulates the TNBC TME and endogenous immune response, its effects may not be substantially enhanced by these ICIs and would better synergize with other ICIs or other immune-based therapies.

To further explore the role of CD8^+^ T cells in limiting pulmonary metastasis, BALB/c mice bearing 4T1-derived tumors were treated for 17 days with either vehicle or Dox, with or without anti-CD8^+^ antibody. Histological analyses of lung tissues revealed increased spontaneous pulmonary metastases in vehicle-treated mice receiving anti-CD8^+^ antibody, while no detectable metastasis was observed in RBΔCDK-activated mice regardless of anti-CD8^+^ treatment (Fig. S6F, G). These findings suggest that RB activation in the tumor compartment limits both tumor progression and metastatic potential, with anti-CD8^+^ treatment exacerbating pulmonary metastasis in the absence of RB activation.

## Discussion

Previous studies and ongoing clinical trials have demonstrated the efficacy of the systemically-acting CDK4/6 inhibitors in treating HR+/HER2- metastatic breast cancer, primarily through the inhibition of RB phosphorylation and subsequent RB activation^11,37,38^. However, multiple TNBC models exhibit redundancy in CDK/Cyclin complexes and display resistance to CDK4/6 inhibition, providing the impetus for combination with CDK2 inhibitors to subvert this redundancy, which has shown promising preclinical efficacy^39,40^. Additionally, while heterogeneous, immunosuppressive TME landscapes across breast cancer subtypes can contribute to the inability of systemic therapies to permeate and act on tumor cells^41,42^. To overcome these phenomena and specifically elucidate the biological functions of tumor-exclusive RB activation, we transfected several TNBC cell lines with a Dox-inducible constitutively active RB allele (RBΔCDK)^33^. Expression of the RBΔCDK construct mimics treatment with a highly tumor-selective cytostatic drug that has no direct effect on the host and enabled us to determine both tumor-intrinsic and extrinsic features of RB activation on cancer biology. This experimental design allowed us to reveal that RB activation solely within the tumor both suppresses intrinsic proliferation and also recruits and activates an anti-tumor CD8^+^ T cell population that limits tumor growth and progression.

In general, resistance to CDK4/6 inhibitors is mediated upstream of RB (e.g., through deregulated phosphorylation)^31^^,^^16^. Thus, the RBΔCDK allele allowed us to determine in resistant TNBC models if there was a downstream perturbation of the RB pathway. In all models tested, including mouse and human TNBC models resistant to palbociclib, RBΔCDK expression was sufficient to induce cell cycle arrest. In contrast, overexpression of wild-type RB did not affect cell proliferation, suggesting that the activation of RB, rather than its mere presence, was key in mediating the cytostatic effect in TNBC. Transcriptomic analysis of palbociclib-resistant TNBC models revealed that RBΔCDK activation downregulated the expression of E2F target genes and genes involved in the G2/M checkpoint, consistent with prior reports^33^.

Induction of gene expression was also observed, and at comparable levels to that observed for gene repression. While this inductive response was more variable, it was generally dominated by genes associated with immunological processes (e.g., IFNγ) or related transcriptional networks (e.g., NFκB). These findings are highly reminiscent of the genes induced with CDK4/6 inhibitor treatment in models and clinical samples that are responsive to CDK4/6 inhibitors^27,30^. Thus, tumor-intrinsic RB activation is sufficient to trigger these responses. While the mechanism of transcriptional repression is likely a feature of direct RB/E2F binding, the features of gene induction are more elusive, as RB has been mapped to bind multiple enhancers and locus control elements that could have far-reaching effects on transcription^15,27,43^. Furthermore, it has been postulated that activation of RB can exert an impact on endogenous retroviruses and other repeat-like elements that can induce interferon-like responses as a feature of viral mimicry^27^.

The main cause of mortality in most cancers is metastatic spread. Here, we find that in both immune competent and deficient models, RBΔCDK was sufficient to suppress metastatic spread. Since RBΔCDK limits the ability of cells to proliferate, it remains unclear if the impact on metastasis is largely a reflection of tumors that fail to proliferate in the metastatic site. Alternatively, RB regulates genes and differentiation states that could limit the metastatic potential of tumor cells. In multiple models, deletion of RB is associated with a more aggressive/invasive phenotype and the progression of ductal carcinoma in situ (DCIS) to invasive breast cancer^44,45^. However, at least in simplistic in vitro migration assays, the expression of RBΔCDK was not sufficient to impede this measure of metastatic biology; thus, the mechanism for the suppression of metastatic progression in this setting remains an area of active investigation. Of note, RB loss or mutation has been observed to be more frequent in metastases as compared to the primary tumor across cancer types and more specifically in HR+ and a small subset of TNBC breast cancer patients^46,47^. Future studies are well-suited to validate these findings using larger patient cohorts.

The sufficiency of tumor-selective RB activation as a therapeutic strategy has not been evaluated previously. Here, in human TNBC xenograft models, RB activation was sufficient to induce potent cytostasis. In the highly aggressive 4T1 model, tumors rapidly bypassed the activation of RB when the tumors were engrafted orthotopically in immunodeficient NSG mice. In immunocompetent BALB/c mice, however, there was a more durable response of 4T1 tumors to RBΔCDK. While AT-3 tumors were expected to behave similarly, they displayed substantially less of a response in immunocompetent mice as compared to 4T1 tumors, potentially due to reduced RBΔCDK expression, less substantial induction of interferon signaling, or a different mechanism altogether. This finding highlights the importance of the immune system, consistent with the variable upregulation of IFN response pathways in TNBC cells in response to RB activation and not simply the expression of human RB.

Consistent with the transcriptional responses and findings in other studies with CDK4/6 inhibitors, RBΔCDK has potent effects on the TME. Specifically, we observed that RB activation in the tumor was sufficient to increase the population of cytotoxic CD8^+^ T cells that promoted anti-tumor activity. Consistent with this finding, anti-CD8 treatment blunted the anti-tumor response of RBΔCDK expression in tumor-bearing BALB/c mice in regard to both tumor size and metastasis. While CDK4/6 inhibition has been shown to enhance the efficacy of anti-PD-1/PD-L1 therapies leading to complete tumor regression in a substantial proportion of animals^48,49^, our data suggest that the immune modulatory effects of tumor-intrinsic RB activation on the TME may not be as potent as those induced by CDK4/6 inhibition. Corroborating this observation is the fact that tumor-intrinsic RB activation resulted in no overt changes to *Pdcd1* or *Ctla4* and even a potential reduction in PD-L1, which would suggest that approaches designed around those targets would prove inconsequential. This finding either supports the significance of the systemic effects of CDK4/6 inhibitors on the host immune compartment (T-cell memory) or that CDK4/6 inhibitors impinge on other features of tumor biology distinct from RB activation^50,51^.

Future studies are well-suited to address some of the limitations in the current study. As mentioned previously, the mechanistic impact of RB activation on metastatic spread requires further investigation. Similarly, RB-activating interventions would likely be less effective if indeed RB is preferentially lost in metastases as compared to the primary tumor. Finally, a more thorough interrogation of the TME upon RB activation by RBΔCDK expression and CDK4/6 inhibitor treatment is necessary. Flow cytometry and cytometry by time of flight (CyTOF) would help resolve the distinct stromal compartments and enable more robust analyses of cell activation states that are introduced in this study through the use of single-cell RNA sequencing and multispectral immunofluorescence.

In conclusion, in certain TNBC settings, tumor-intrinsic activation of RB was effective to reprogram the TME, thus crosstalking in a tumor-extrinsic manner to engage the endogenous adaptive immune system to limit disease progression. While additive or synergistic anti-tumor effects were not observed in concert with anti-PD-L1 or anti-CTLA-4 blockade, future detailed studies are warranted to explore combinations with other immunotherapies, such as other ICIs (alone or in combination), vaccines, and/or cell therapies, for potentially enhanced therapeutic benefit.

## Methods

### Cell culture

All cell lines were incubated at 37 °C with 5% CO₂ and tested to be mycoplasma-free. Human primary TNBC cell lines HCC1806 (CRL-2335, RRID:CVCL_1258) and MDA-MB-231 (HTB-26, CVCL_0062) were obtained from ATCC and were grown in Dulbecco’s Modified Eagle’s Medium (DMEM) (Corning, 10-013-CV) and Roswell Park Memorial Institute (RPMI) 1640 medium (Corning, 10-040-CV) supplemented with 10% Fetal Bovine Serum (FBS) (Gibco, A52567-01) and 1% antibiotic-antimycotic (Gibco, 15240-062). Mouse TNBC cell lines AT-3 (RRID: CVCL_VR89) and 4T1 (RRID: CVCL_0125) were obtained from Dr. Scott Abrams (Roswell Park Comprehensive Cancer Center, RPCCC) and were grown in DMEM and RPMI 1640 media supplemented with 10% FBS and 1% antibiotic-antimycotic, respectively. All cell lines were tested and found to be mycoplasma-free based on DAPI staining and polymerase chain reaction (PCR). The MDA-MB-231 and HCC1806 cell lines were authenticated using short tandem repeat (STR) profiling. All cell culture experiments were carried out within ten passages following recovery of the cells from the cell bank.

### Cloning, transfection, and infection

FLAG-tagged RBΔCDK constructs were amplified by PCR from the corresponding pcDNA3 constructs and were transferred to the pINDUCER20 cloning vector, as described previously^32,33^. The pINDUCER20 lentiviral construct was packaged in HEK293FT cells by transfection. AT-3, 4T1, HCC1806 and MDA-MB-231 cells were infected with the lentiviral particles in the presence of 5 μg/mL polybrene (Sigma Aldrich). The infected cells were selected by single cell cloning, treated with dimethyl sulfoxide (DMSO) or Doxycycline (Dox) (Sigma, D9891), and validated by immunofluorescence (IF), as described previously^52^.

### Cell proliferation assay

The live-cell imaging system IncuCyte S3 was employed to monitor cell growth. The cell lines were transduced to express H2B-GFP, and measurements of GFP+ cells were conducted to assess proliferation in real time. Fold change in cell number was normalized to baseline measurements. Dose-response curves were produced by graphing the drug dose versus cell growth to determine the IC_50_.

### Western blotting

Primary antibodies for western blotting analysis included RB (4H1) (Cell Signaling, 9309L), pRB (S807/811) (Cell Signaling, 8516S), Cyclin A1 (Sigma, C4710), β-Actin (R&D Systems, MAB8929), and Flag (Proteintech, 20543-1-AP). The cells were lysed in RIPA lysis solution with 1X protease inhibitor (Thermo Fisher, 1860932) and 1 mM Phenylmethanesulfonylfluoride (PMSF) (Sigma) to create whole-cell extracts. After 30 μg of extracted proteins were separated using SDS-PAGE, they were transferred onto Nitrocellulose Pure Transfer Membrane (Santa Cruz, sc-3724). After 30 minutes of blocking by 5% milk in TBST and an overnight incubation at 4 °C with primary antibodies, membranes were treated for up to an hour at room temperature with horseradish peroxidase (HRP)-tagged anti-mouse (Santa Cruz, sc-516102), anti-rabbit (Invitrogen, A27036), or anti-goat (Santa Cruz, sc-2354) secondary antibodies, as described previously^37^. Thermo Fisher’s enhanced chemiluminescence kit (34096) was utilized to identify immunoreactive bands.

### Immunofluorescence

TNBC cells were seeded on glass coverslips, washed with 1X PBS, fixed in methanol for 5 minutes, and permeabilized with 0.5% Triton X-100 for 10 minutes. Next, the cells were blocked with IF buffer (1X PBS, 5% BSA, 0.4% NP40) and incubated with the primary antibody, RB (4H1), at 37 °C for 1 hour. Following primary antibody incubation, the coverslips were washed with PBS and incubated with donkey anti-mouse IgG Alexa Fluor 594 secondary antibody (Invitrogen, A21203) along with DAPI for nuclear staining. After the secondary antibody incubation, the coverslips were washed again with PBS and mounted onto glass slides. Images were captured using an EVOS fluorescence microscope at 40X magnification.

### Trans-well migration assay

1 × 10⁵ cells were seeded into the upper chamber of an 8 μm pore membrane using serum-free media based on the corresponding cell line. The lower chamber was filled with media containing 20% FBS. After 24 hours of incubation, residual cells on the upper membrane were removed using cotton applicators. The cells that migrated through the membrane were fixed in 3.7% formaldehyde (Sigma) prepared in PBS, stained with 0.1% crystal violet, and then photographed and counted using an EVOS fluorescence microscope at 20X magnification.

### 3D culture

50 μL of 50% Matrigel basement layer was used to pre-coat a 96-well plate and was allowed to solidify for 20 minutes at room temperature. TNBC cell lines tagged with H2B-GFP were seeded at a density of 2000 cells per well in 96-well plates. Using live-cell imaging, the seeded cells were exposed to 1 μg/mL of Dox and were allowed to grow for four to six days to produce 3D cultures.

### Mice and cell line-derived tumors

RPCCC’s animal care facilities housed all female NSG and BALB/c mice used in this study. Corresponding to the NIH guidance for the care and use of laboratory animals, all animal care, drug treatments, and sacrifices were authorized by the Institutional Animal Care and Use Committee (IACUC) of RPCCC. Mammary fat pad injections of 1 × 10^6^ cells per mouse were used to produce 4T1 tumors in both NSG and BALB/c mice. Mammary fat pad injections of 3 × 10^6^ cells per mouse were used to produce AT-3 tumors in both NSG and C57BL6NCrl mice. Mouse mammary fat pad injections of 3 × 10^6^ cells each were used to produce HCC1806 and MDA-MB-231 xenografts in NSG mice. Mice were randomized to treatment. Dox water was prepared by combining 0.5 g Dox and 2.5 g sucrose (Sigma, S0389) in 250 mL of water. The formula for calculating tumor volume was (length*width*width)/2. The tumors were embedded in paraffin and examined further after the mice were sacrificed. Mouse body mass was periodically measured to monitor adverse effects. After 16-30 days of treatment, mice were euthanized by CO_2_ and cervical dislocation. Mice were excluded from tumor weight endpoint analyses if they weren’t sacrificed at the same timepoint.

### Histological analysis

Freshly cut tumor tissues and excised lungs were processed and paraffin-embedded following fixation in 10% neutral formalin solution (VWR). The embedded tissues were serially sectioned at a thickness of 4–6 µm. The slides were dried at 65 °C for 2 hours, then deparaffinized on the BOND RXm Research Stainer (Leica Biosystems) using BOND Dewax solution (AR9222, Leica Biosystems). Immunohistochemical (IHC) DAB staining was performed with the BOND Polymer Refine Detection Kit (Leica Biosystems, #DS9800) using the automated staining protocols programmed into the BOND RXm. After processing, slides were preserved with glass coverslips and Cytoseal XYL mounting medium (Epredia, #8312-4). CD8 (Abcam, ab209775), Ki67 (Abcam, ab16667), and RB (4H1) antibodies were used for IHC staining. Hematoxylin and Eosin (H&E) staining was performed on the sectioned tissues. Images were acquired using the Vectra Polaris Instrument and analyzed with QuPath software. Pulmonary metastases were defined by heterogeneous appearance and were validated by an in-house pathologist (AKW). Histological analysis was performed in a blinded manner.

### Multispectral immunofluorescence (mIF) staining

The formalin-fixed paraffin-embedded (FFPE) tissue sections were stained by multispectral immunofluorescence (mIF) using the Opal 6-Plex Detection kit (AKOYA Bioscience, NEL821001KT), as described in our previous study^53^. The antibodies included in the mIF panel were DAPI, Pan Keratin (Wide Spectrum cytokeratin, Abcam, Opal 480), CD8 (Abcam, ab209775, Opal 570), PD-L1 (64988, Cell Signaling, Opal 650), CD3 (ab16669, abcam, Opal 520), CD11c (97585, Cell Signaling, Opal 780), CD163 (ab182422, abcam, Opal 540), and FOXP3 (12653, Cell Signaling, Opal 690). The PhenoImager HT® Automated Quantitative Pathology Imaging System (AKOYA Biosciences) was utilized to image the slides. AKOYA Biosciences inForm® Software v2.6.0 was used to analyze the slides in an unbiased fashion.

### Transcriptome analysis

In order to initially determine an RNA Integrity Number (RIN), RNA was extracted using the Qiagen RNeasyplus kit and examined using the Agilent 2200 TapeStation (Agilent) while using the RNA6000 Nano assay. In this study, only samples with RIN > 7.0 were included. To provide a full-length, strand-specific representation of nonribosomal RNA transcripts, cDNA synthesis was carried out utilizing random hexamers. The DriverMap Human Genome-Wide Gene Expression Profiling Sample Prep Kit hDM18Kv3 (Cellecta Inc.) was used to prepare targeted RNA sequencing libraries. When oligonucleotides were ligated by PCR amplification, standard Illumina adaptors were inserted along with sample-specific “barcodes” that flanked the target sequence. This allowed for dual-index sequencing and the deconvolution of sample-specific reads using standard Illumina software. In summary, anchor PCR was carried out with a 5-minute hot start at 95 °C, 15 cycles of 95 °C – 0.5 min, 68 °C – 1 min, and 72 °C – 1 min, and a final 10-minute extension at 72 °C to reduce primer dimer formation. To evaluate replicability, the reaction products were verified in triplicate on an agarose gel. Following SPRI (Agentcourt, 1:1 sample: reagent ratio) purification, the PCR products were measured using the Qubit fluorescence assay (Qubit dsDNA HS Assay Kit, Thermo Fisher Scientific). Using a NextSeq500/550 High Output v2 Kit (75 cycles) and an Illumina NextSeq 500 sequencer, target-enriched RNAseq libraries were examined in accordance with the manufacturer’s protocol (Illumina). For bulk RNA-sequencing, raw sequencing reads from AT-3 and 4T1 cells treated with Dox as well as DMSO-treated controls were used as input for the nf-core/rna-seq pipeline (version 3.3) using mm10 as the reference genome. Differentially expressed genes between cells treated with Dox and DMSO from each of the two cell lines were identified using DESeq2 (version 1.28.0) with raw sequence counts produced from the above RNA-Seq processing pipeline as inputs. Genes extracted with adjusted *p* values < 0.05 and Log_2_ fold change > 1 or < −1 were deemed as significantly altered due to treatment.

### Gene set enrichment analysis

For each cell line, differential gene expression analysis was conducted between RBΔCDK-activated and control samples using the DESeq2 software package. The Log_2_-fold change values were used as input for gene set enrichment analysis (GSEA) preranked analysis, which calculated the normalized enrichment scores (NES) and corresponding *p* values for each of the 50 hallmark gene sets from the Molecular Signature Database (MSigDB, v2022.1. Hs). This analysis was performed using the fgsea Bioconductor package. The NES values and their associated adjusted *p* values for each cell line were combined into a bubble chart, visualizing the NES and adjusted *p* values across gene sets and cell lines. Gene sets were ordered based on the NES values observed in the indicated cell lines.

### 10X single-cell RNA sequencing

Liberase (Sigma, 05401020001) was used to digest tumors removed from 4T1 RBΔCDK tumors treated with vehicle (*n* = 5) or Dox (*n* = 5), resulting in single cells. Ten thousand single cells were then sent to RPCCC’s Genomics Shared Resources for sequencing using the 10X Chromium and Illumina NovaSeq instruments, per their recommended number of cells for single-cell analysis (www.kb.10xgenomics.com). Input files with the name “filtered_feature_bc_matrix.h5” generated from the cellranger software (version 6.1.2, 10X Genomics) for the control and Dox-treated samples were used as input for analysis using the Seurat (version 4.2.0) R package. Single cells were filtered by setting the following parameters: minFeature = 200, MaxFeature = 4000, maxPercentMT = 10 (8 for Dox-treated samples) and maxPercentLargestGene = 20 (15, for Dox-treated samples). After filtering, 7338 cells remained in the control group and 9419 cells remained in the treatment group. For quantitative comparisons of the two groups, the treatment group was down-sampled by randomly selecting 7338 cells to match the same number of cells in the control group. The two datasets were then integrated with the following workflows: Normalize data, FindVariableFeatures (selection.method = ‘vst, nfeatures = 2000), SelectIntegrationFeatures (nfeatures = 3000), FindIntegrationAnchors, IntegrateData. The DefaultAssay was then set to “integrated” and the following workflows were performed: ScaleData, RunPCA (npcs = 30), then ElbowPlot was used to identify the optimal principal component (PC) numbers to use for downstream commands. 20 was found to be the optimal PC number and was used to set the “dims” parameter for the following commands: RunUMAP, FindNeighbors. Lastly, FindClusters was run by setting “resolution = 0.5”. Finally, single cell clusters were annotated with scType^54^.

### Immune cell depletion/neutralization

For the first depletion, 0.5 mg of anti-CD8 antibodies (BioXCell, BE0004-1) were injected intraperitoneally one day before Dox treatment per mouse. For the subsequent depletions, 0.2 mg of anti-CD8 antibodies were injected twice per week per mouse^55^.

### Statistical analysis

Kaplan-Meier curves were plotted for mouse overall survival using the survival (version 3.6.4) and survminer (version 0.4.9) packages from base R (version 4.3.0). Statistical tests used and levels of significance are defined in each corresponding figure legend and include student’s *t*-test for endpoint comparisons, two-way ANOVA for analyses with two variables, and log-rank tests for survival analyses.

## Supplementary information

Supplementary Information

## Data Availability

All data generated in this study are available from the corresponding author upon reasonable request. The data discussed in this publication have been deposited in NCBI's Gene Expression Omnibus^56^ (PMID: 11752295) and are accessible through GEO Series accession numbers GSE310025 and GSE310026.

## Code availability

The code used for the single-cell RNA sequencing data has been deposited at the following location: https://github.com/jianxinwang/Collections/blob/main/scRNA-Seq/scRNA-Seq_Analysis_4T1_RBdCDK.R.
