# Supplementary material for: Intrinsic RB activation induces tumoral and stromal anti-tumor responses that limit triple-negative breast cancer
Source: NPJ Breast Cancer. 2025 Dec 1;11:134. doi: 10.1038/s41523-025-00845-5 (PMC12669681; doi:10.1038/s41523-025-00845-5)
Supplement: Supplementary file 1 — Supplementary Information [file 41523_2025_845_MOESM1_ESM.pdf]

Supplementary Figure 1:

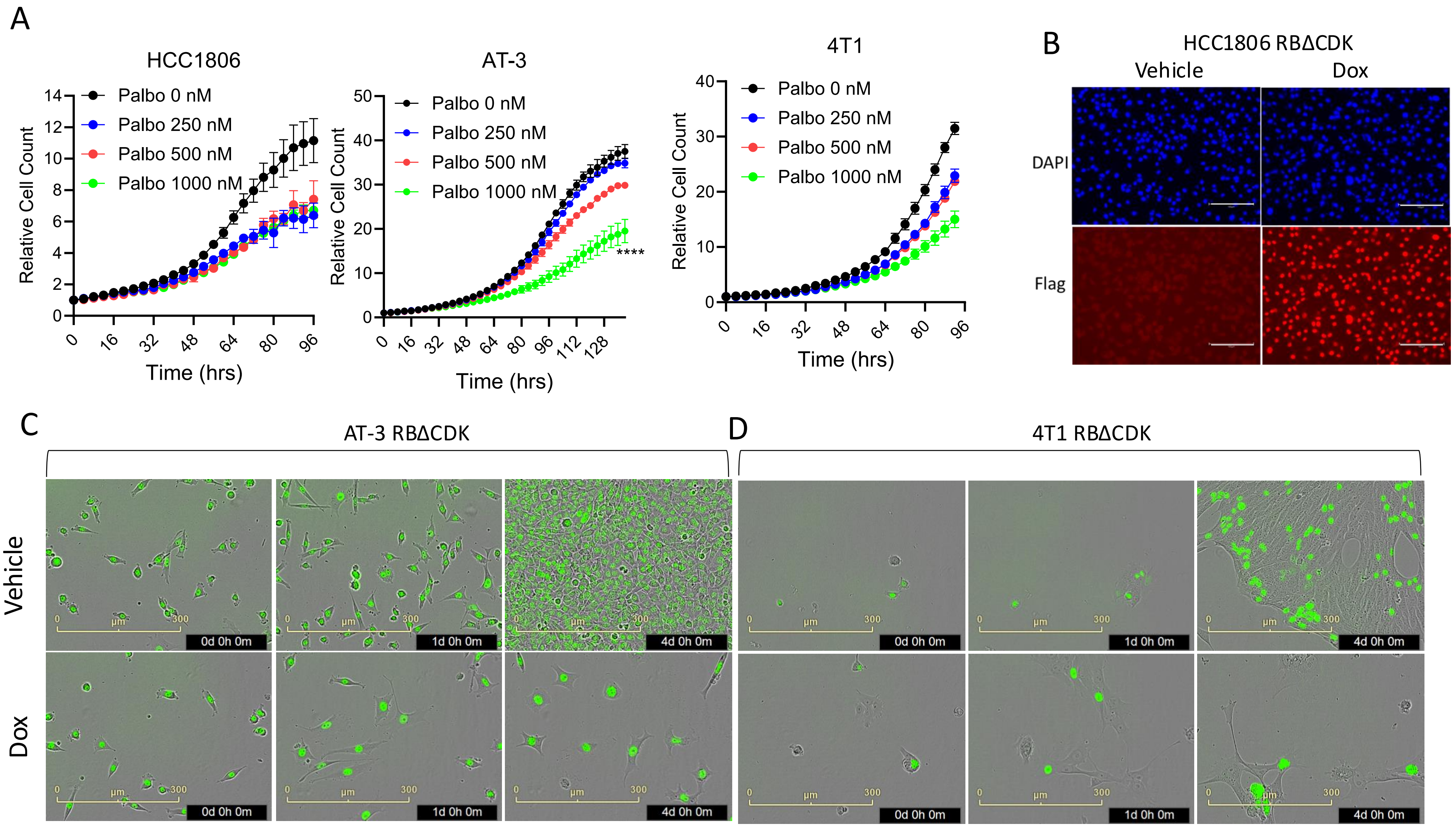

Supplementary Figure 1:

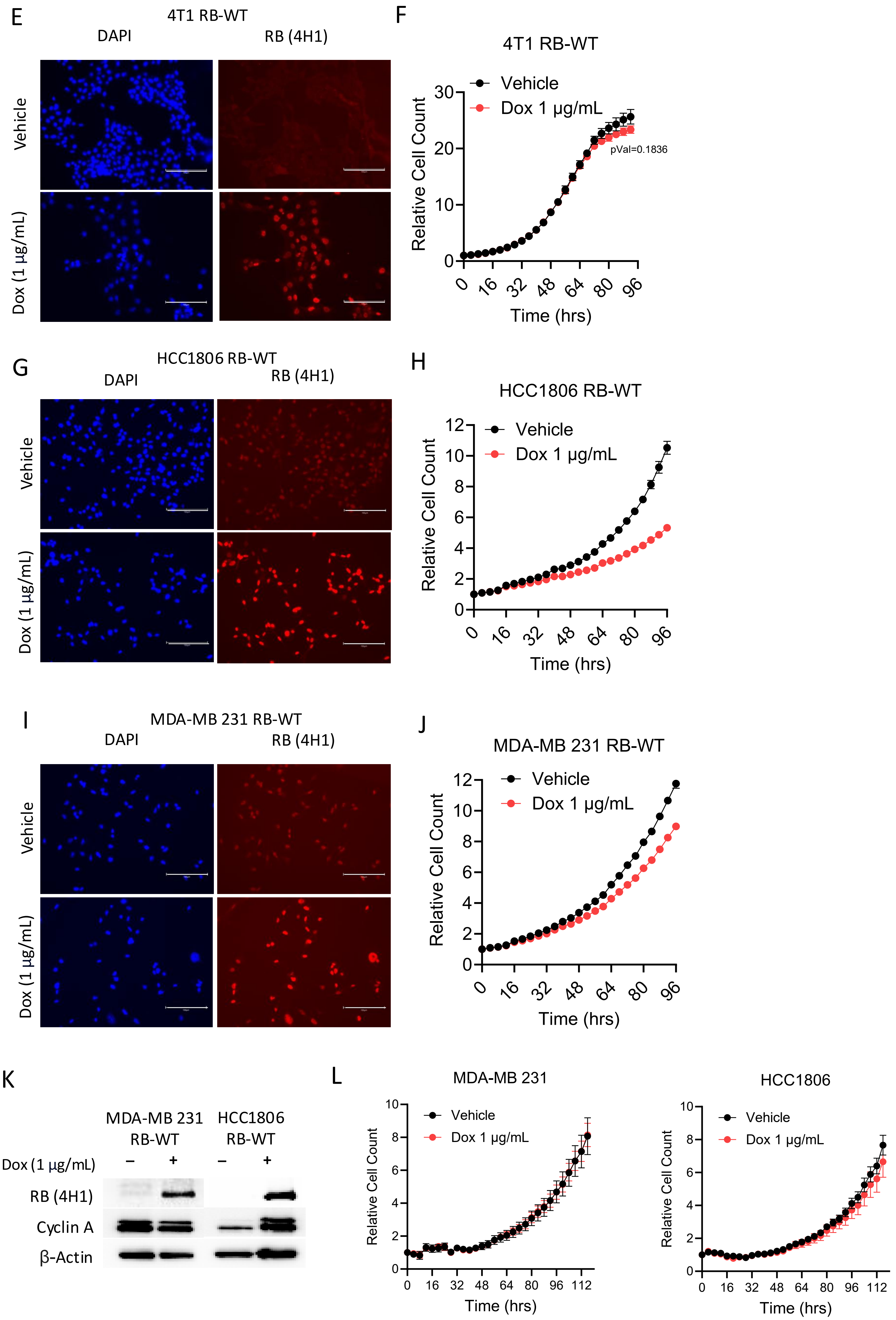

Supplementary figure 1. RB activation inhibits cell growth in palbociclib-resistant TNBC cell lines. (A) Live cell imaging of the indicated TNBC cell lines treated with different concentrations of palbociclib. (B) Immunofluorescence images of DAPI (blue) and Flag (red) expression in HCC1806 RBΔCDK cells treated with vehicle or doxycycline (Dox) (1 μg/mL). Scale bar = 75 μm. (C-D) Live cell image stills on the indicated RBΔCDK cell lines treated with vehicle or Dox (1 μg/mL). Scale bar = 300 μm. (E-J) Immunofluorescence images of DAPI (blue) and wildtype RB (RB-WT, red) in Dox-inducible RB-WT transfected 4T1, HCC1806 and MDA-MB 231 cells treated with vehicle or Dox (1 μg/mL) and live cell imaging proliferation curves. Scale bar = 150 μm. Data displayed as mean +/- SEM. (K) Immunoblotting of the indicated RB-WT human cell lines in the presence and absence of Dox. (L) Live cell imaging of the indicated TNBC human cell lines treated with vehicle or Dox (1 μg/mL). Data reported as mean +/- SEM, n = 6 for each live-cell cohort.

Supplementary Figure 2:

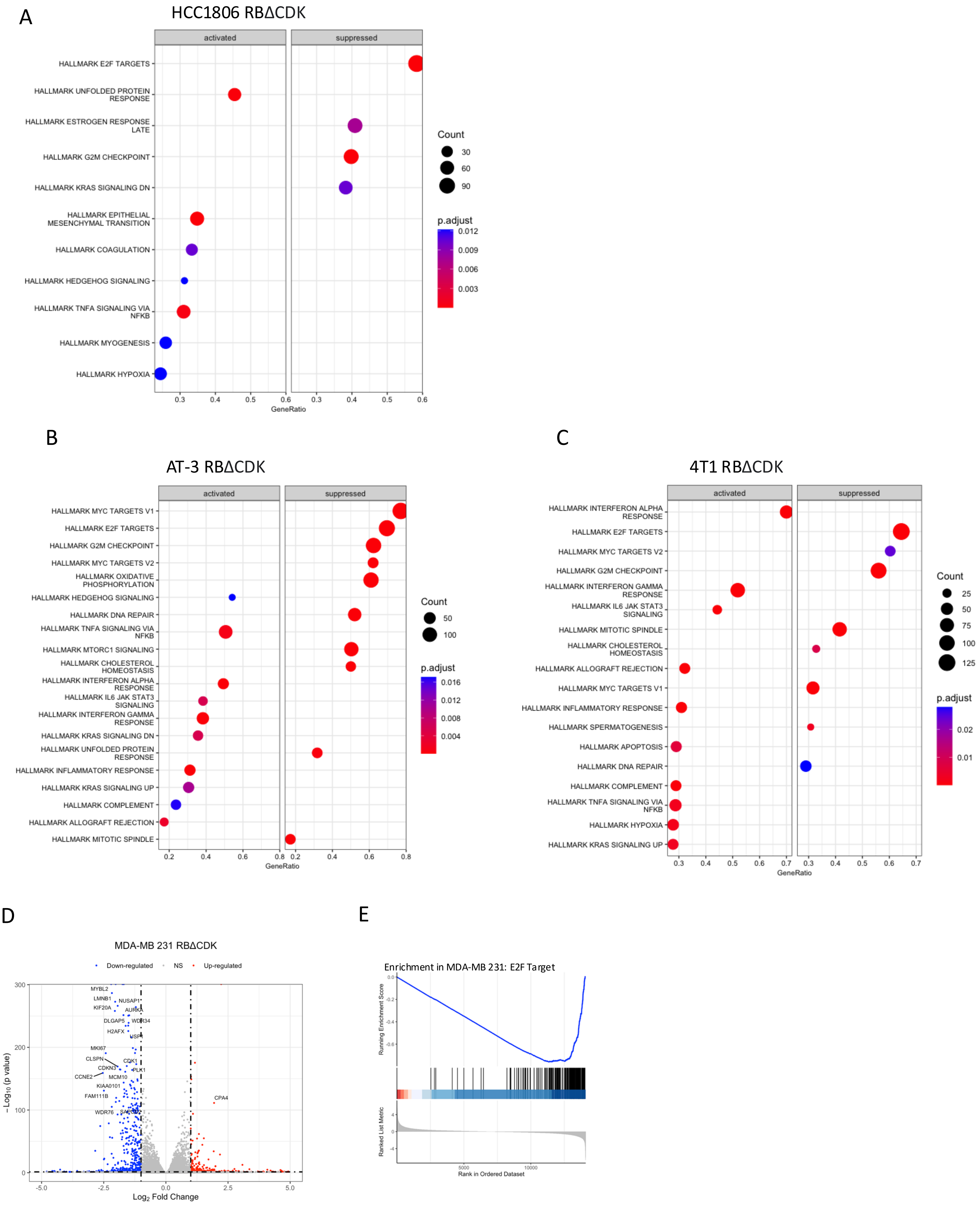

Supplementary figure 2. RB activation impacts transcriptional programs in TNBC cell lines. (A-C) Bubble plots generated from RNA sequencing data of the most significantly altered pathways following RBΔCDK activation in the indicated cell lines. (D) Volcano plots indicating the differentially expressed genes based on transcriptome analysis in the MDA-MB-231 RBΔCDK cell line treated with Doxycycline (Dox) (1 µg/mL) for 48 hours compared to non-treated cells. Blue represents genes that were significantly downregulated, red represents genes that were significantly upregulated. (E) Gene set enrichment analysis (GSEA) of the E2F Target pathway from the MDA-MB-231 cell line following RBΔCDK activation.

Supplementary Figure 3:

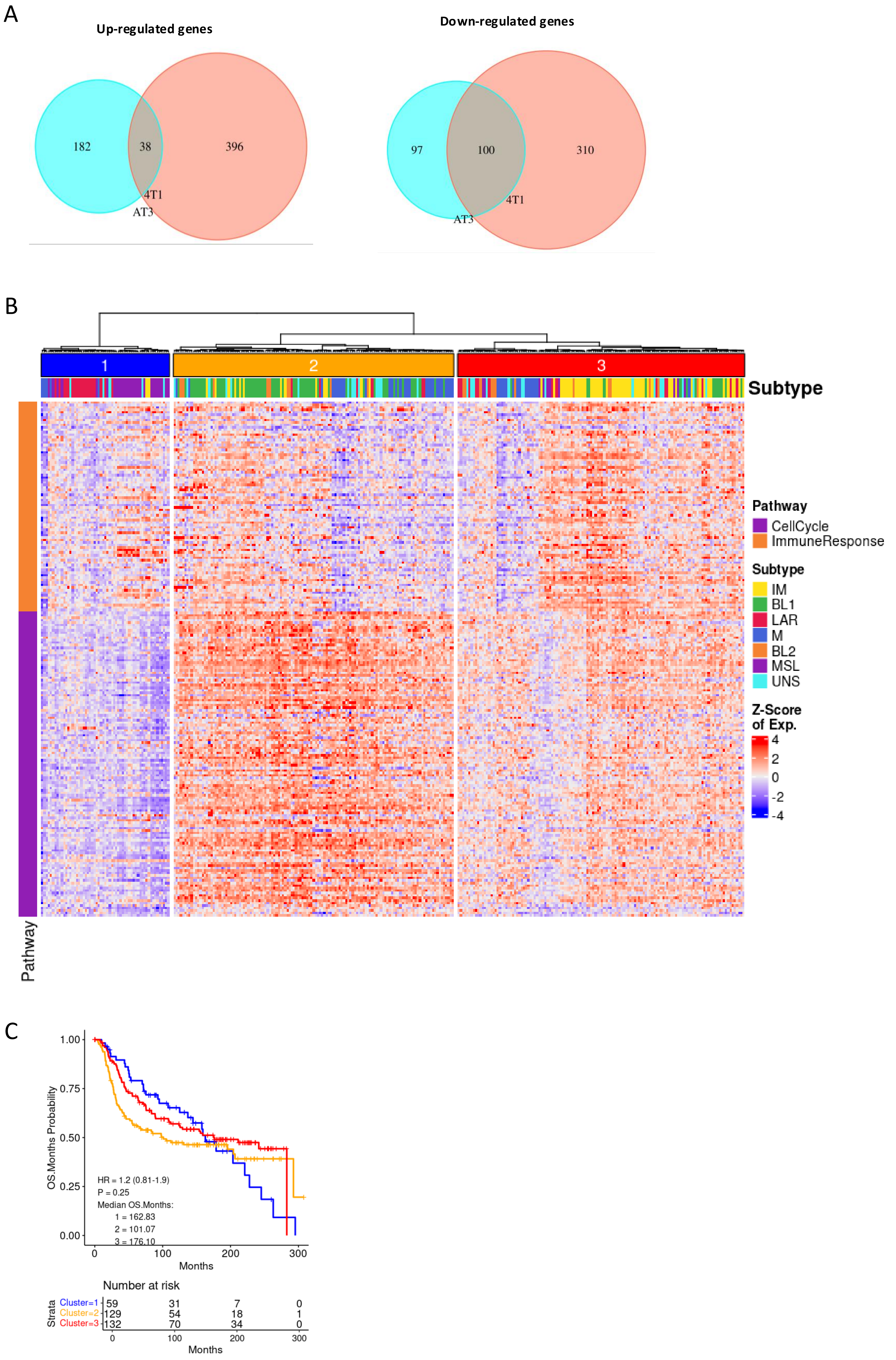

Supplementary figure 3. Clinical impact of RB activation. (A) Venn diagram showing the overlap of upregulated and downregulated genes following RBΔCDK activation in both AT-3 and 4T1 cell models. (B) Heatmap depicting the expression of immune response– and cell cycle–related genes regulated by RB activation across the METABRIC breast cancer dataset. (C) Kaplan–Meier survival analysis of patient clusters identified in B, illustrating differences in overall survival based on RBΔCDK-associated gene expression profiles.

Supplementary Figure 4:

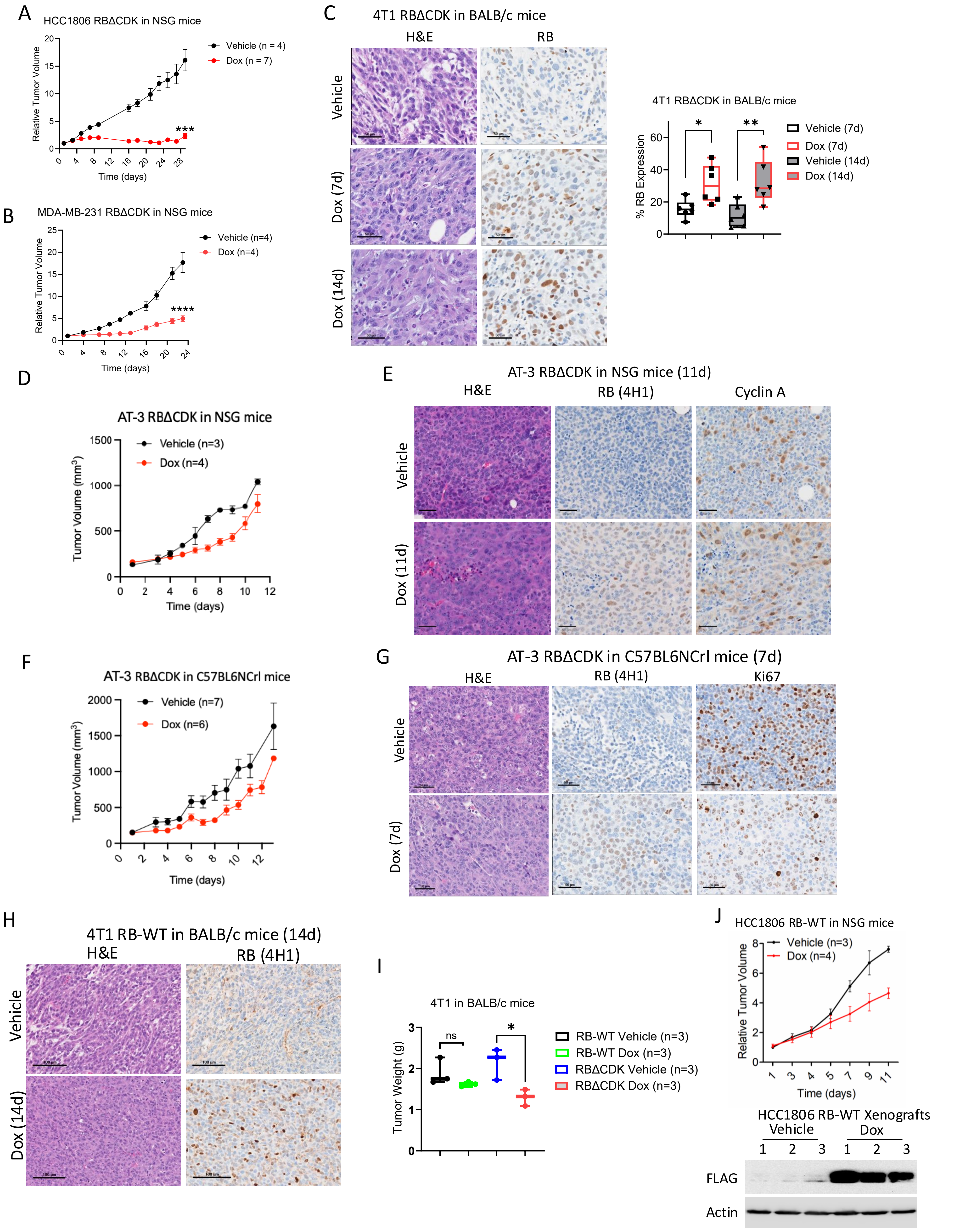

Supplementary figure 4. TNBC 3D culture and tumor growth is blunted upon RB activation. (A) Relative tumor volume of HCC1806 RBΔCDK and (B) MDA-MB-231 RBΔCDK xenografts in NSG mice following vehicle or doxycycline (Dox) water (2 mg/mL) treatment. *** $p<0.001$, **** $p<0.0001$ as determined by two-tailed t test, data displayed as mean +/- SEM. (C) Hematoxylin and eosin (H&E) and immunohistochemical (IHC) staining to determine the RBΔCDK expression in 4T1 tumors in BALB/c mice under 7 days and 10 days of Dox treatment. Box and whisker plots indicating the quantification of RBΔCDK expression in the indicated tumors. Scar bar = 50 µm. * $p<0.05$, ** $p<0.01$ as determined by two-tailed t test. (D) Tumor volume of AT-3 RBΔCDK xenograft in NSG mice following vehicle or doxycycline (Dox) water (2 mg/mL) treatment. (E) H&E and IHC staining indicating the RBΔCDK and Cyclin A expression in AT-3 tumors in NSG mice under 11 days of Dox treatment. Scar bar = 50 µm. (F) Tumor volume of AT-3 RBΔCDK xenograft in C57BL6NCrl mice following vehicle or doxycycline (Dox) water (2 mg/mL) treatment. (G) H&E and IHC staining to indicate the RBΔCDK and Ki67 expression in AT-3 tumors in C57BL6NCrl mice under 7 days of Dox treatment. Scar bar = 50 µm. (H) H&E and IHC staining depicting human RB expression in Dox-inducible RB-WT transfected 4T1 tumors engrafted into BALB/c mice after 14 days treated with vehicle or Dox water (2 mg/mL). Scar bar = 100 µm. (I) Box and whisker plots of tumor mass at 14 days of RB-WT and RBΔCDK-transfected 4T1 tumors in BALB/c mice following vehicle and Dox water (2 mg/mL) treatment. * $p<0.05$ as determined by two-way ANOVA. (J) Relative tumor volume of HCC1806 RB-WT xenograft in NSG mice following vehicle or doxycycline (Dox) water (2 mg/mL) treatment. Immunoblotting of the indicated RB-WT xenografts treated with vehicle or Dox water.

Supplementary Figure 5:

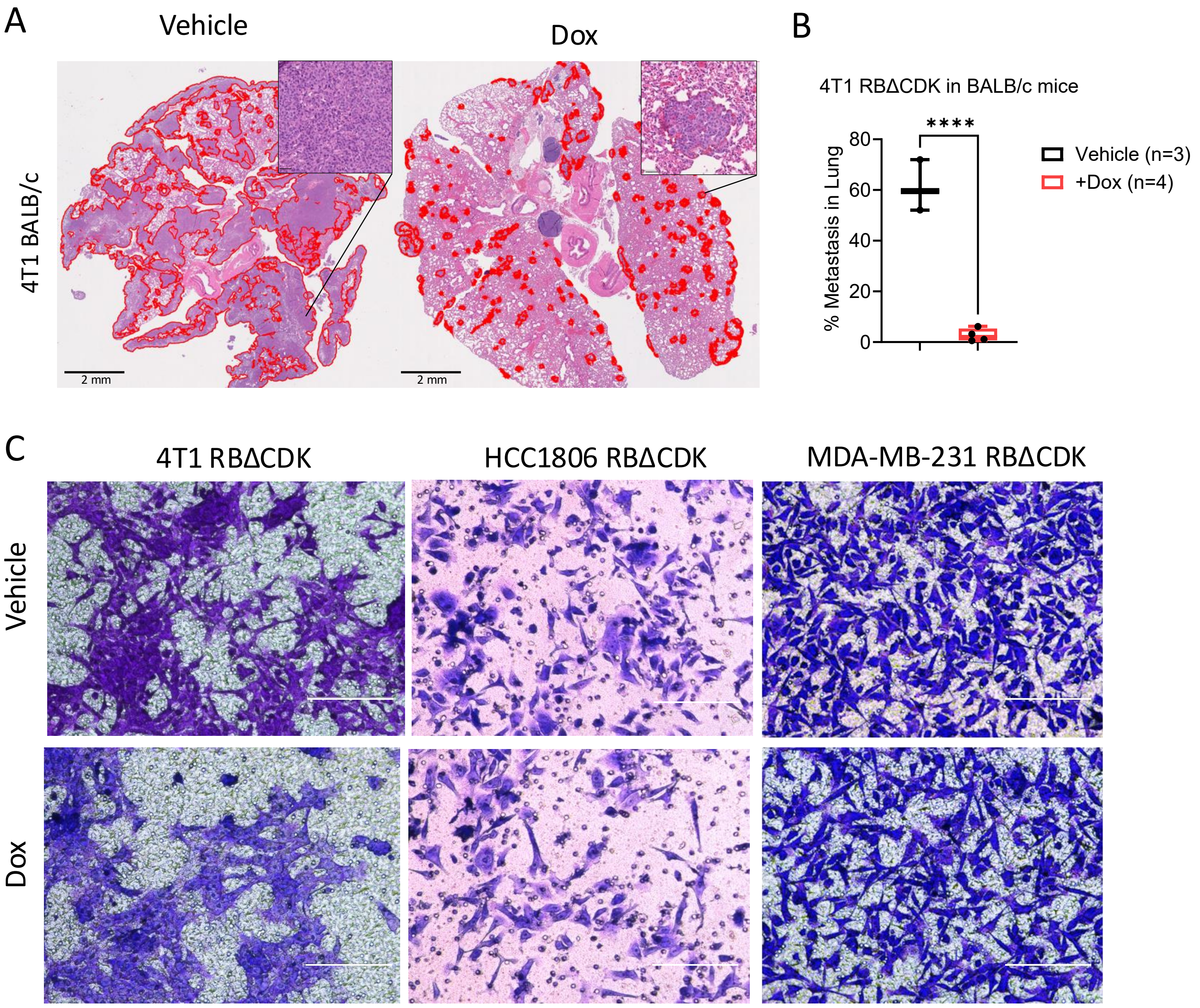

Supplementary figure 5. RB activation limits metastatic potential of TNBC xenografts. (A) Representative images of hematoxylin and eosin (H&E) staining of lung tissues excised from BALB/c mice implanted with RBΔCDK 4T1 cells via tail injection that were treated with vehicle or doxycycline (Dox) water (2mg/mL) for 10 days. Red demarcations denote metastasis nodules. Scale bar = 2 mm and 50 μm. (B) Box and whisker plots of pulmonary metastasis in mice from (A). **** $p<0.0001$ as determined by two-tailed t test. (C) Trans-well migratory assay of the indicated RBΔCDK cell lines following vehicle or Dox (1 μg/mL) treatment. Scale bar = 150 μm.

Supplementary Figure 6:

A

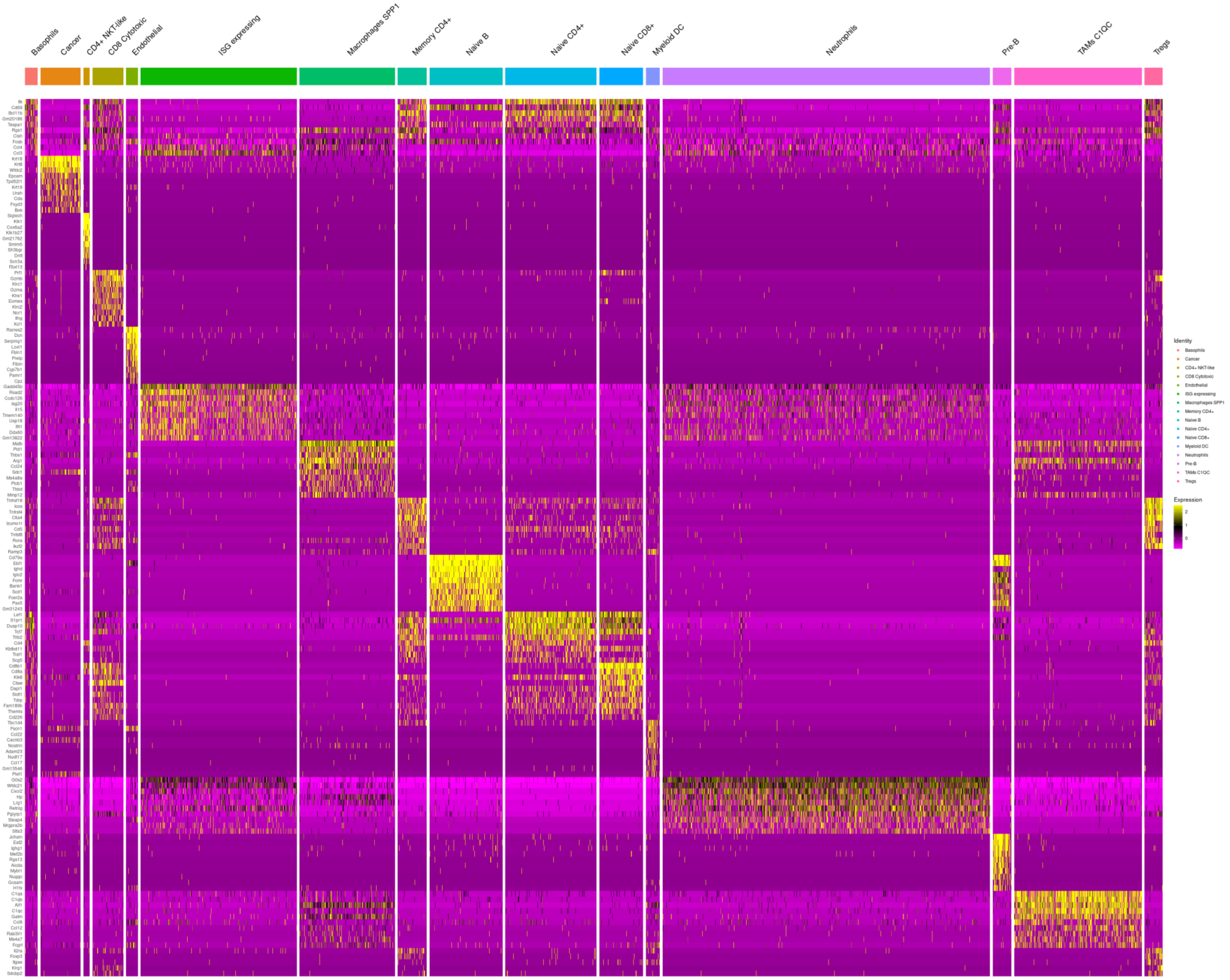

Supplementary Figure 6:

B

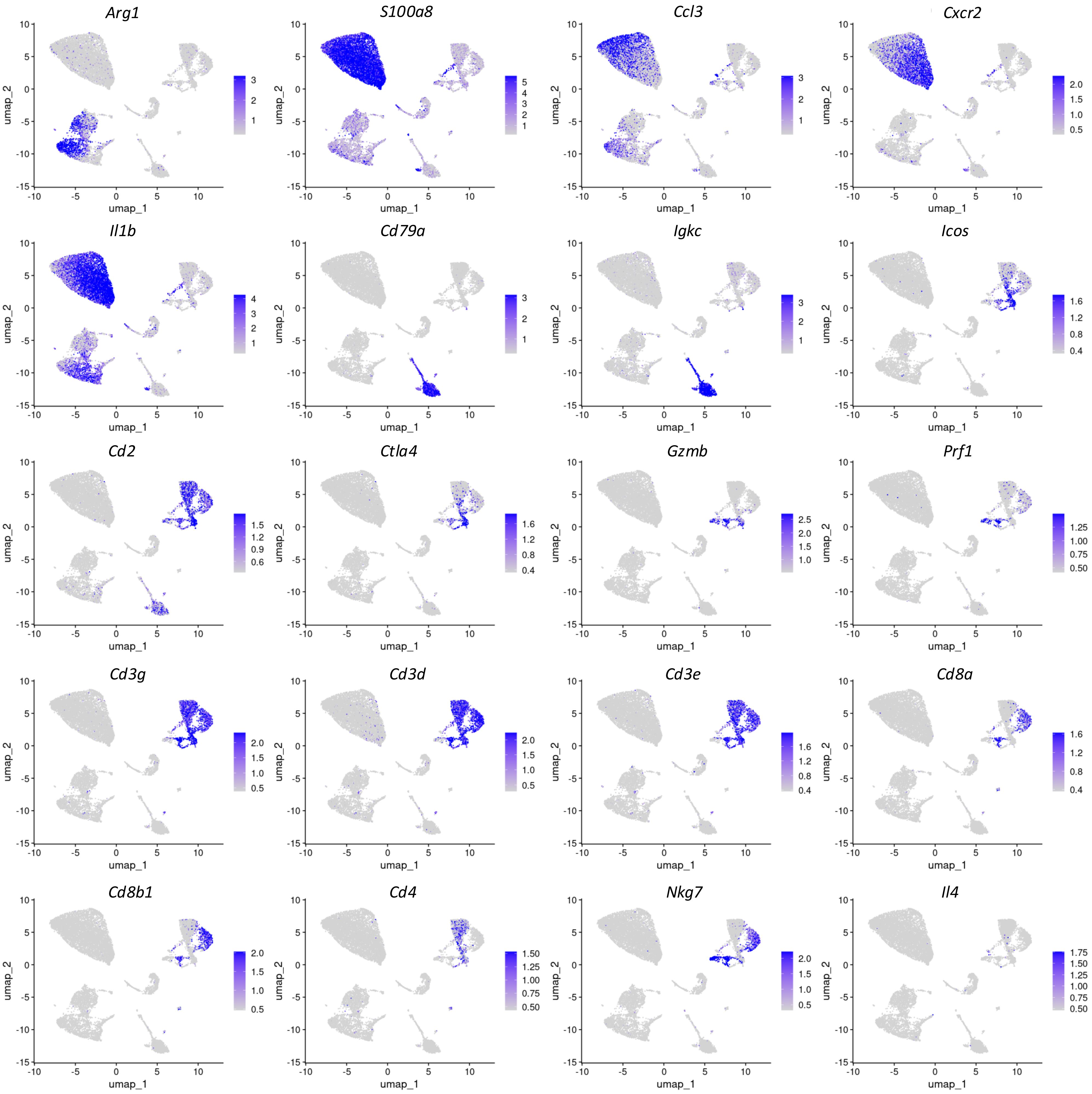

Supplementary Figure 6:

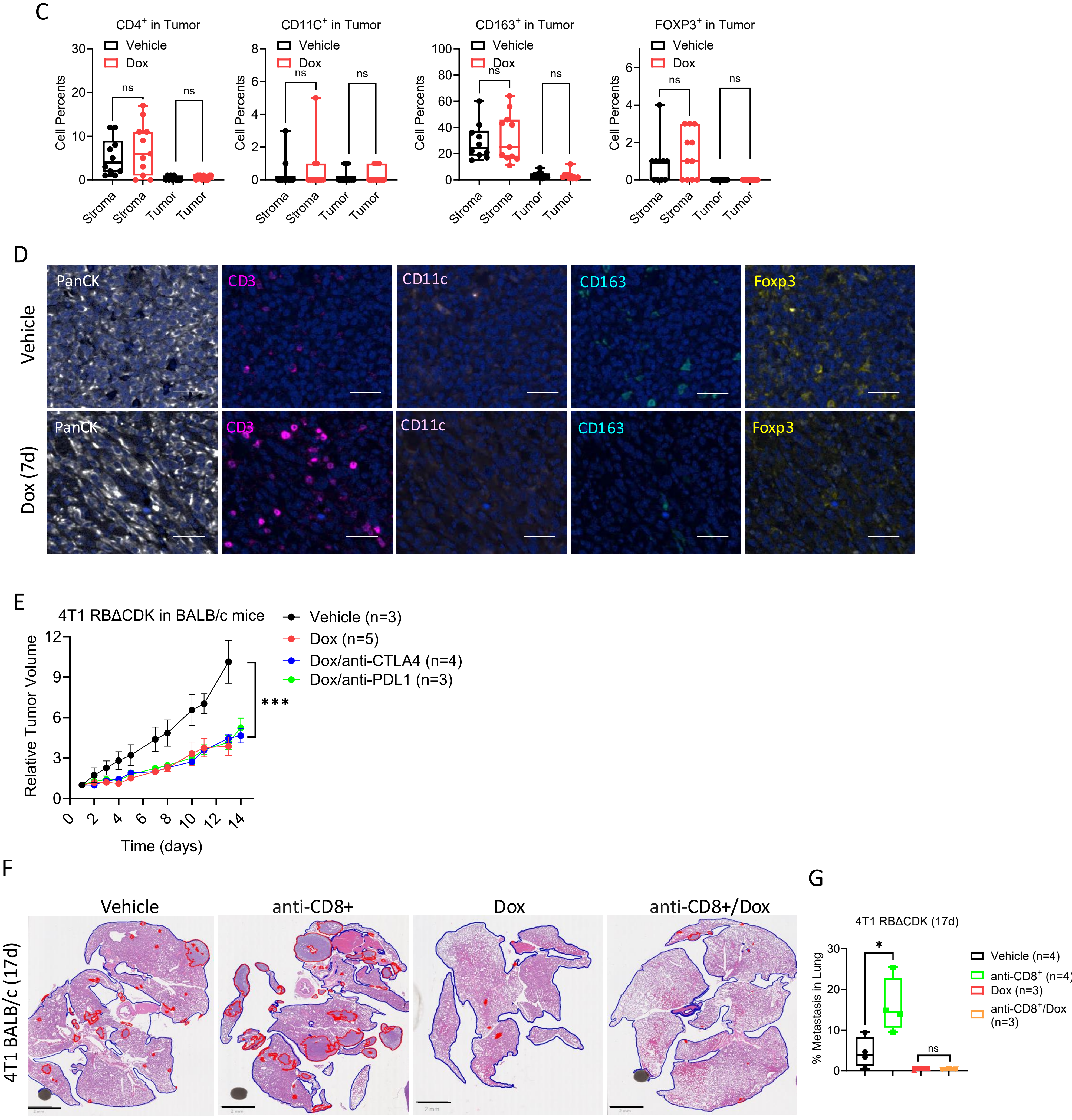

Supplementary figure 6. RB activation in TNBC tumor models alters the tumor microenvironment. (A) Seurat heatmap depicting the expression of genes that distinguish different clusters that represent different cellular components comparing RBΔCDK 4T1 tumors in BALB/c mice treated with doxycycline (Dox) water (2mg/mL) to vehicle-treated mice for 6 days. (B) Seurat feature plot indicating the differential expression of representative cluster markers. (C) Representative multispectral immunofluorescent (mIF) images of RBΔCDK 4T1 tumors from BALB/c mice treated with either vehicle or Dox water (2 mg/mL) for 7 days stained for pan-cytokeratin (PanCK, white), CD3 (dark pink), CD11c (light pink), CD163 (cyan), FOXP3 (yellow) and DAPI (blue). Scale bar = 50 μm. (D) Box and whisker plots quantifying the indicated mIF image staining from tumors in C in both the tumor and stromal compartments. ** $p<0.01$, *** $p<0.001$ as determined by two-tailed t test, n ≥ 3 mice per condition, with analysis from 3-5 regions of interest (ROIs) per mouse. (E) Relative tumor volume of RBΔCDK 4T1 tumors in BALB/c mice after 17 days following treatment with vehicle, Dox water (2 mg/mL), anti-CTLA4 antibody, anti-PDL1 antibody, alone or in combination. *** $p<0.001$ as determined by two-way ANOVA. (F) Representative hematoxylin and eosin (H&E) images of lung tissues excised from BALB/c mice implanted with RBΔCDK 4T1 tumors treated with vehicle, Dox water, anti-CD8 antibody, or the combination for 17 days. (G) Box and whisker plots of lung metastases from (F). * $p<0.05$ as determined by two-way ANOVA.

Western blot raw data

Figure 1A and 1B

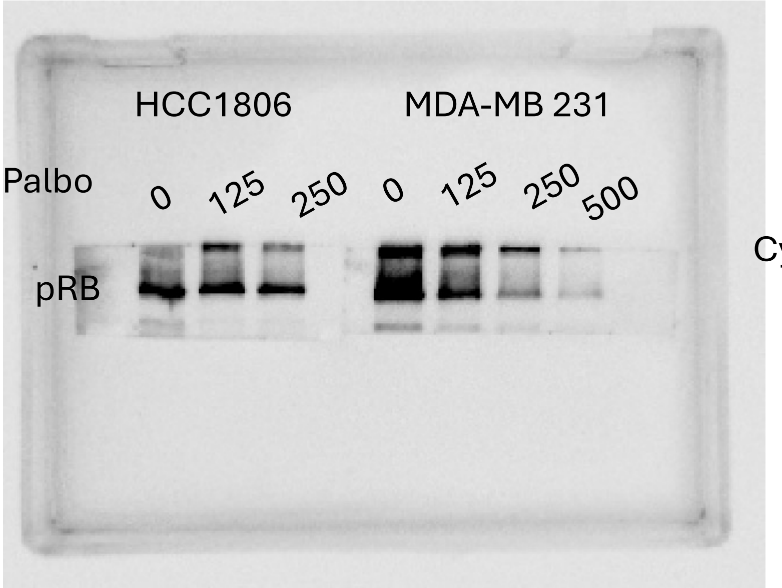

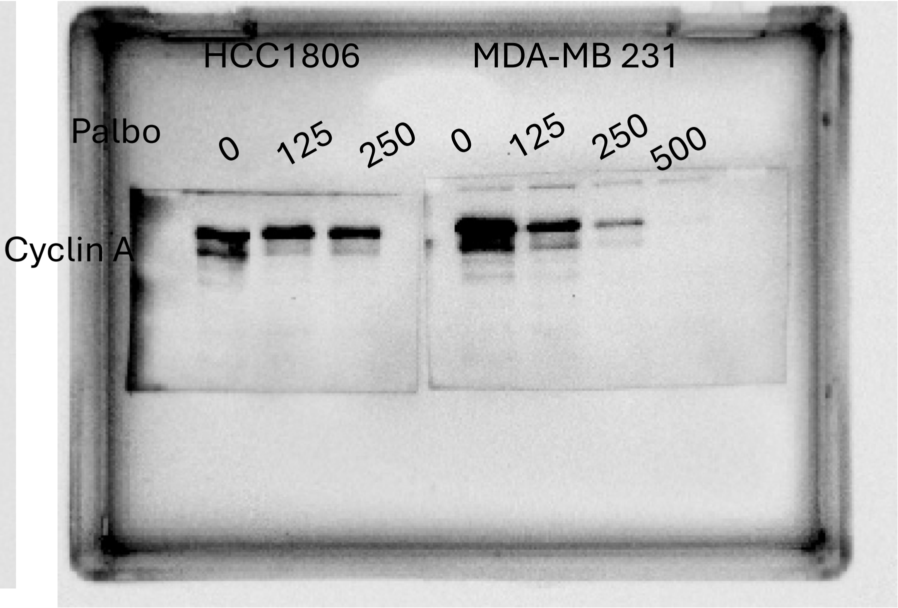

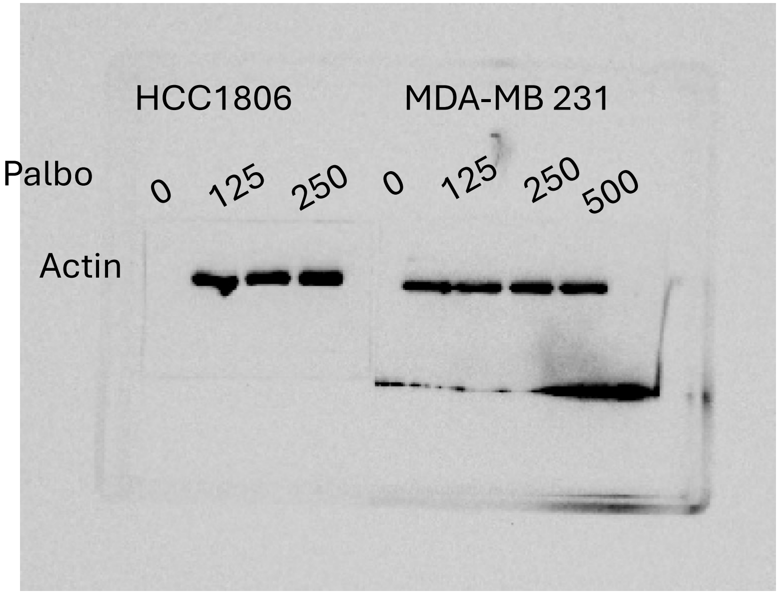

Figure 1C and 1D

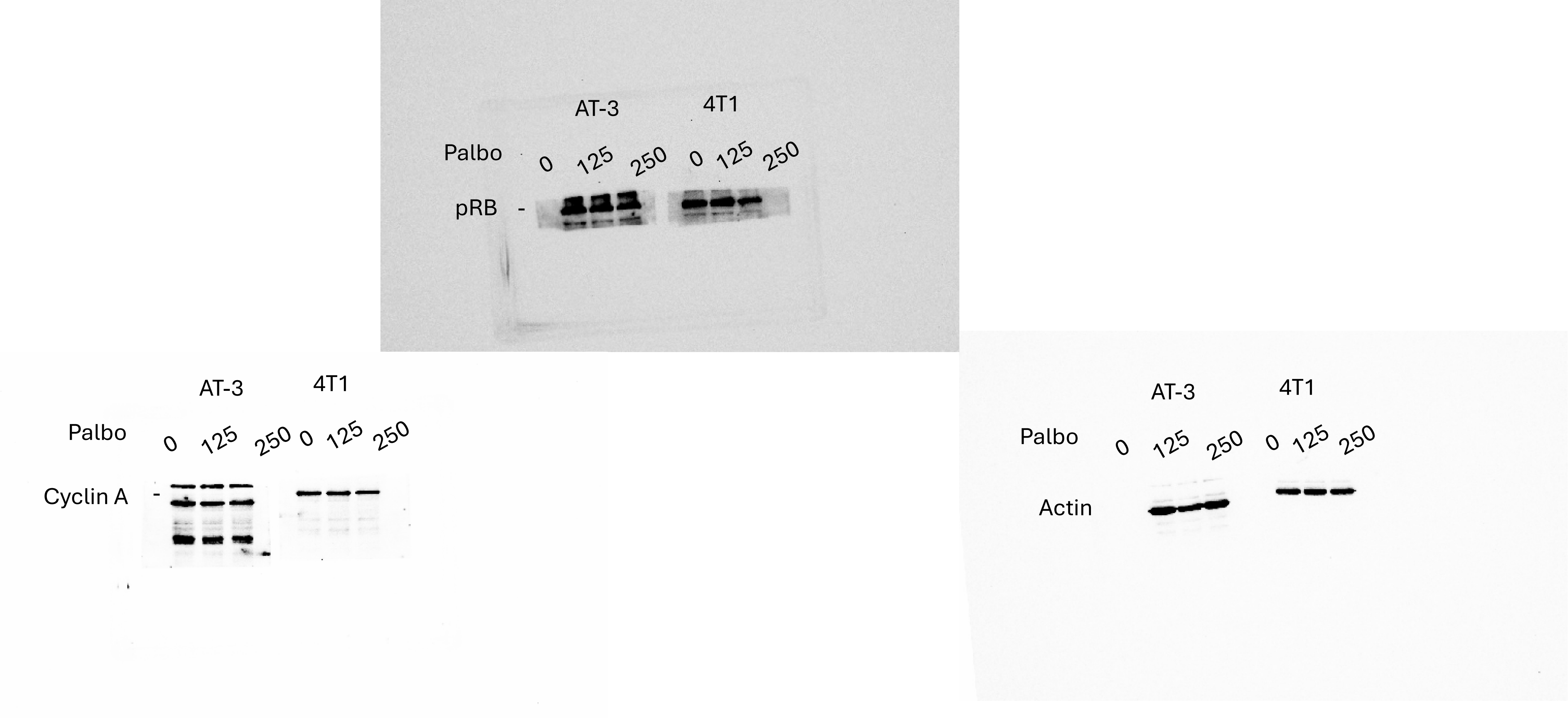

Figure 1F

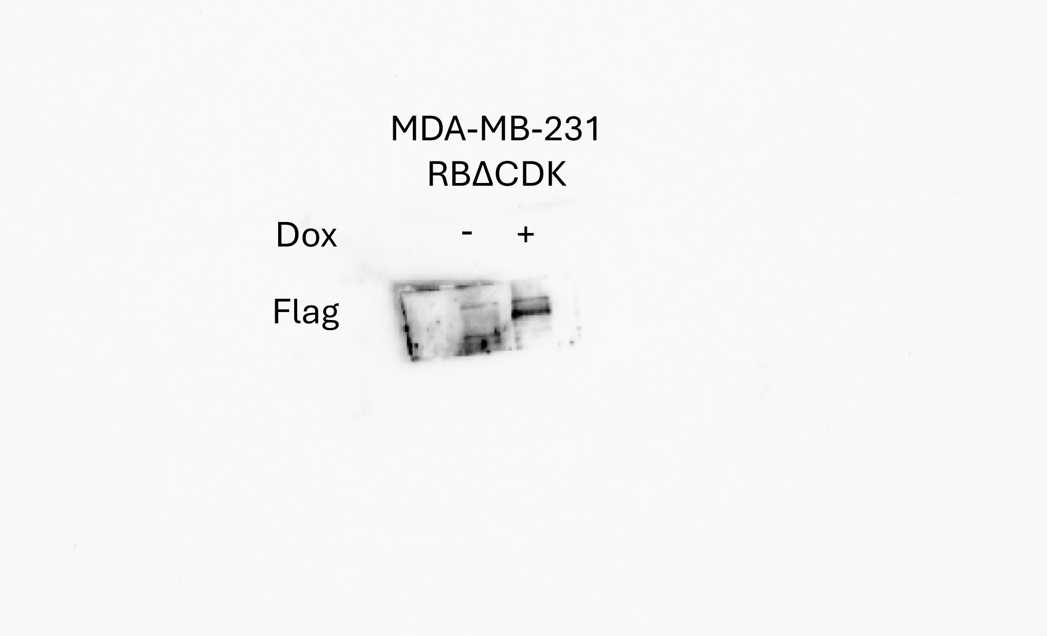
MDA-MB-231
RBΔCDK
Dox
-
+
Flag

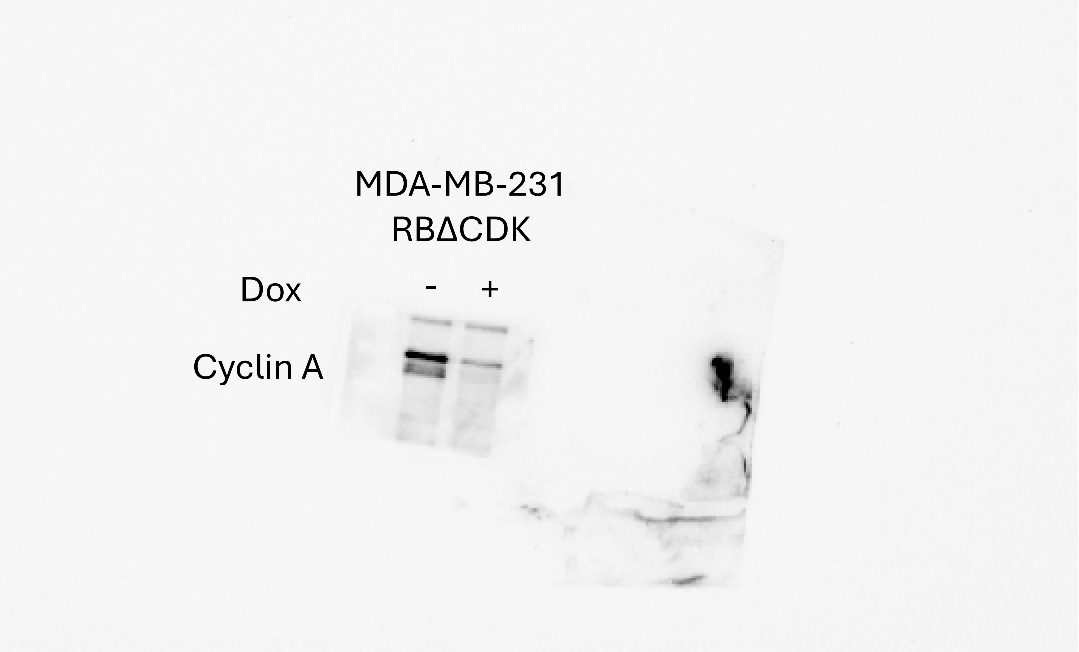
MDA-MB-231
RBΔCDK
Dox
-
+
Cyclin A

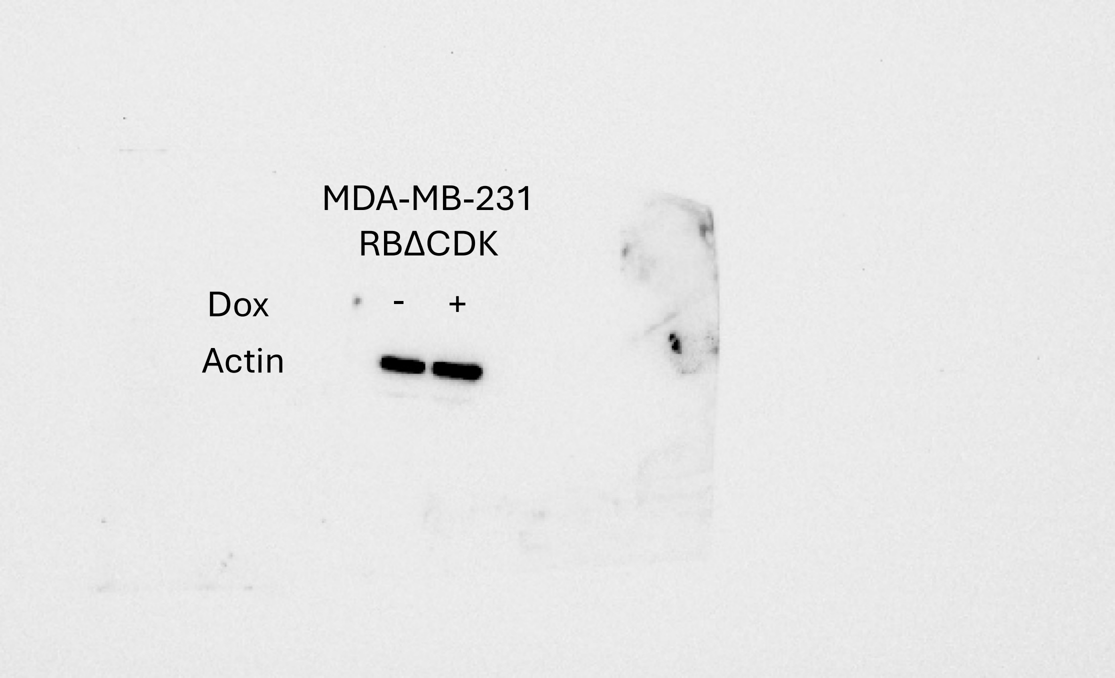
MDA-MB-231
RBΔCDK
Dox
-
+
Actin

Figure 1F

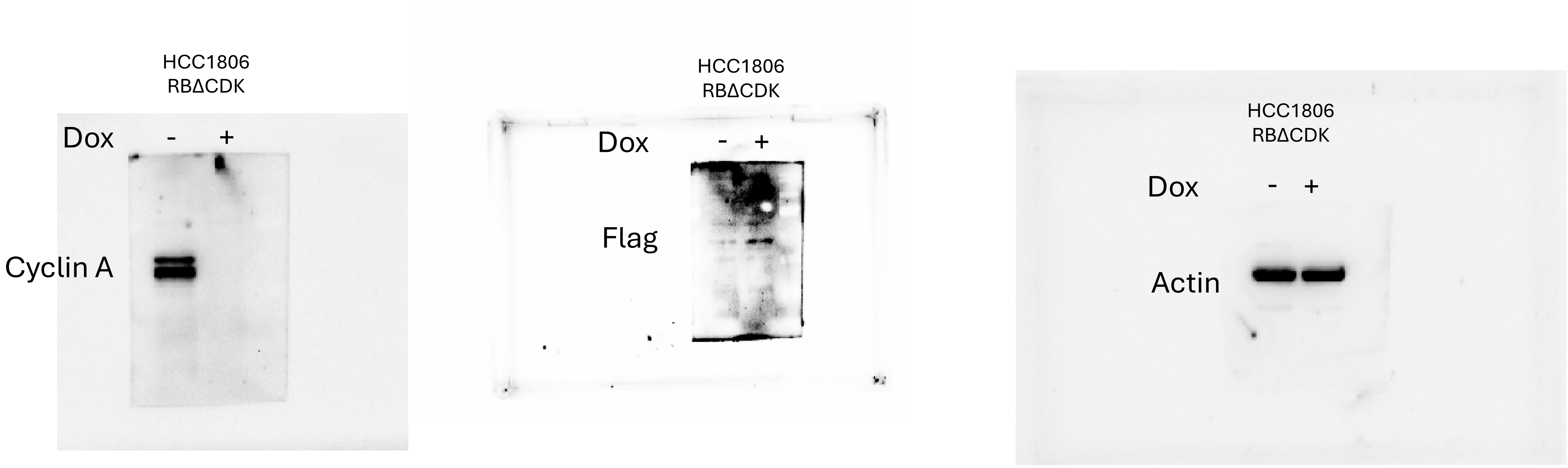
HCC1806
RBΔCDK
Dox - +
Cyclin A
HCC1806
RBΔCDK
Dox - +
Flag
HCC1806
RBΔCDK
Dox - +
Actin

Figure 1H

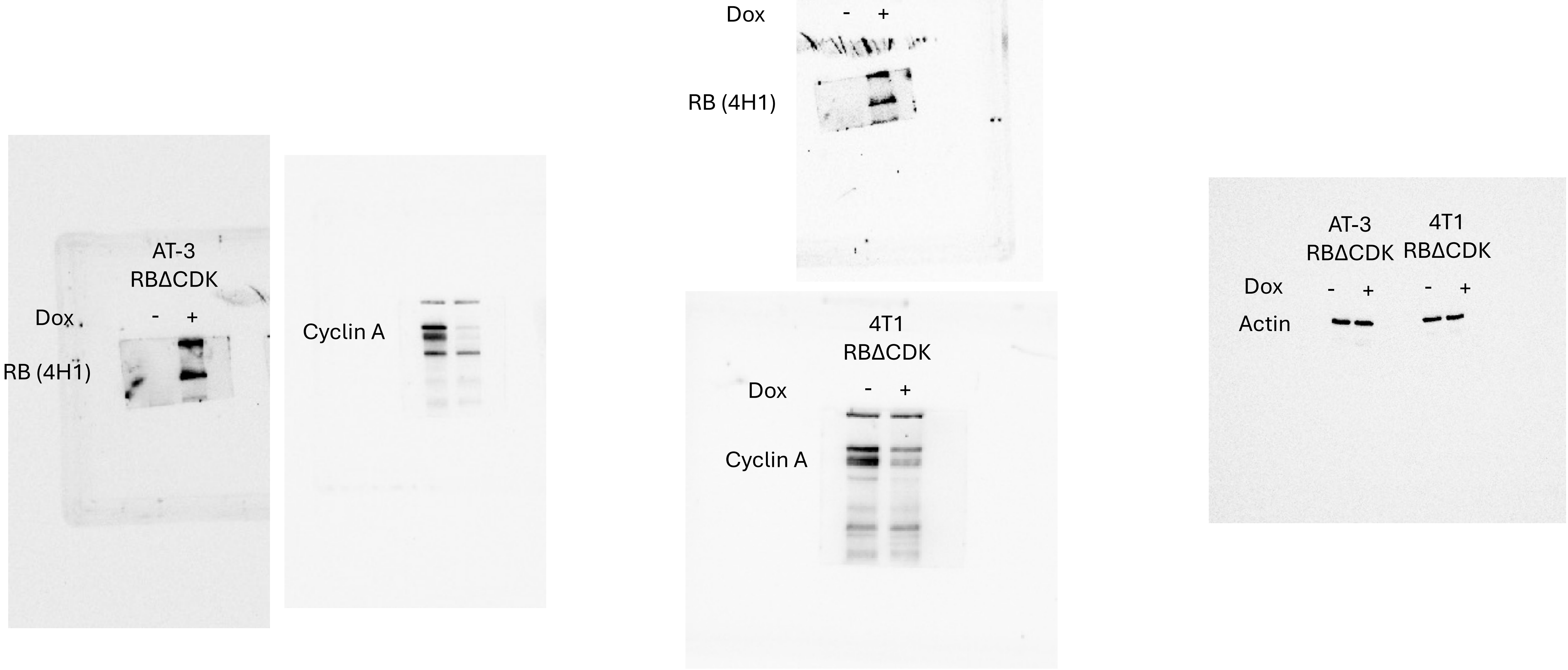

AT-3
RBΔCDK
Dox
- +
RB (4H1)
Cyclin A
4T1
RBΔCDK
Dox
- +
RB (4H1)
4T1
RBΔCDK
Dox
- +
Cyclin A
AT-3
RBΔCDK
4T1
RBΔCDK
Dox
- + - +
Actin

Figure S1K

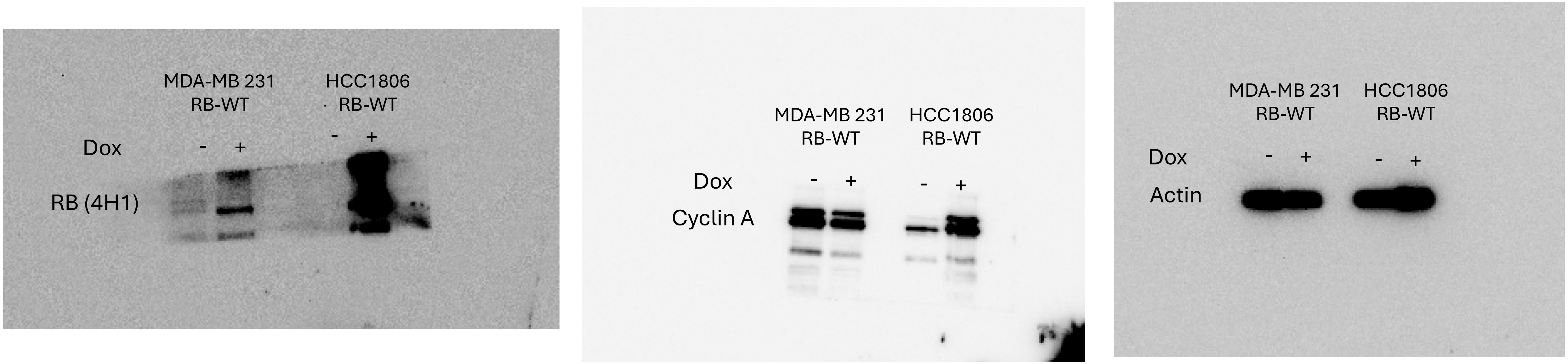
MDA-MB 231 RB-WT
HCC1806 RB-WT
Dox - + - +
RB (4H1)
MDA-MB 231 RB-WT
HCC1806 RB-WT
Dox - + - +
Cyclin A
MDA-MB 231 RB-WT
HCC1806 RB-WT
Dox - + - +
Actin

Figure S4J

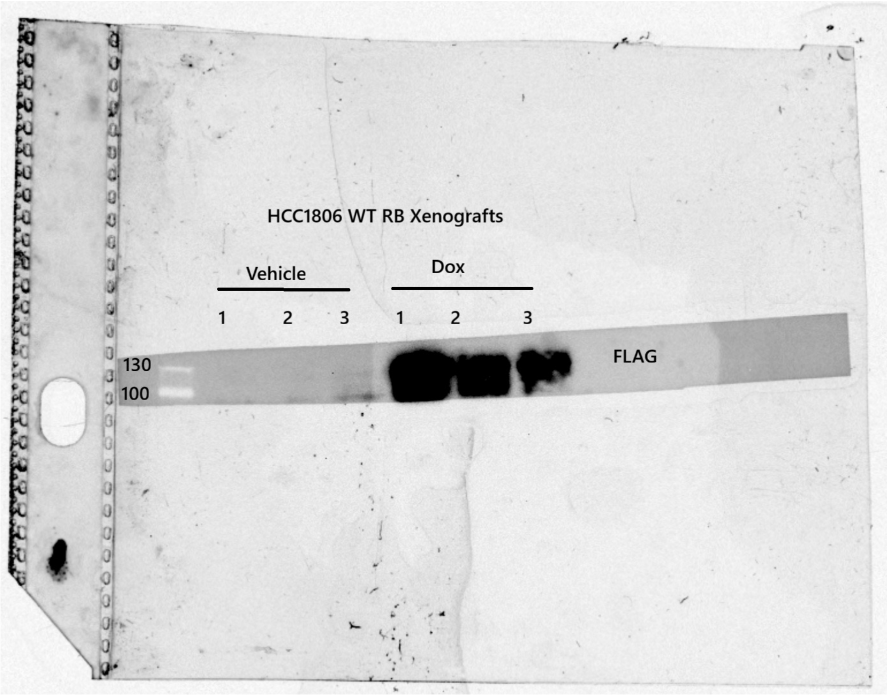
HCC1806 WT RB Xenografts
Vehicle
Dox
1
2
3
1
2
3
130
100
FLAG

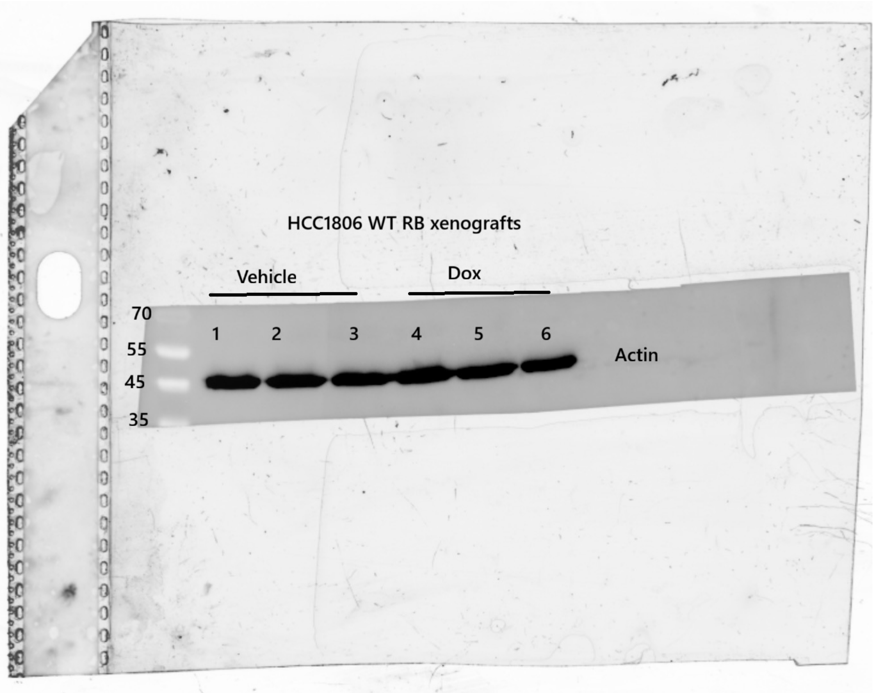
HCC1806 WT RB xenografts
Vehicle
Dox
70
55
45
35
1
2
3
4
5
6
Actin
